# Amidinatotetrylenes Donor Functionalized on Both N Atoms: Structures and Coordination Chemistry

**DOI:** 10.1021/acs.inorgchem.3c04135

**Published:** 2024-01-30

**Authors:** Christian Alonso, Javier A. Cabeza, Pablo García-Álvarez, Rubén García-Soriano, Enrique Pérez-Carreño

**Affiliations:** † Departamento de Química Orgánica e Inorgánica, Centro de Innovación en Química Avanzada ORFEO−CINQA, Universidad de Oviedo, E-33071 Oviedo, Spain; ‡ Departamento de Química Física y Analítica, Universidad de Oviedo, E-33071 Oviedo, Spain

## Abstract

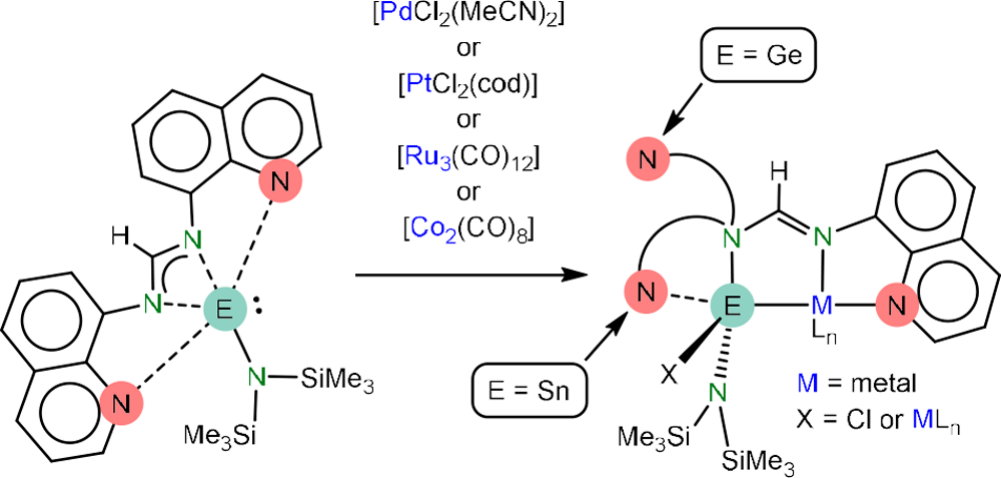

E(hmds)(bqfam) (E = Ge (**1a**), Sn (**1b**); hmds = N(SiMe_3_)_2_, bqfam = *N,N′-bis*(quinol-8-yl)formamidinate), which are amidinatotetrylenes equipped with quinol-8-yl fragments on the amidinate N atoms, have been synthesized from the formamidine Hbqfam and Ge(hmds)_2_ or SnCl(hmds). Both **1a** and **1b** are fluxional in solution at room temperature, as the E atom oscillates from being attached to the two amidinate N atoms to being chelated by an amidinate N atom and its closest quinolyl N atom (both situations are similarly stable according to density functional theory calculations). The hmds group of **1a** and **1b** is still reactive and the deprotonation of another equivalent of Hbqfam can be achieved, allowing the formation of the homoleptic derivatives E(bqfam)_2_ (E = Ge, Sn). The reactions of **1a** and **1b** with [AuCl(tht)] (tht = tetrahydrothiophene), [PdCl_2_(MeCN)_2_], [PtCl_2_(cod)] (cod = cycloocta-1,5-diene), [Ru_3_(CO)_12_] and [Co_2_(CO)_8_] have been investigated. The gold(I) complexes [AuCl{κ*E*-E(hmds)(bqfam)}] (E = Ge, Sn) have a monodentate κ*E*-tetrylene ligand and display fluxional behavior in solution the same as that of **1a** and **1b**. However, the palladium(II) and platinum(II) complexes [MCl{κ^3^*E,N,N*′**-ECl(hmds)(bqfam)}] (M = Pd, Pt; E = Ge, Sn) contain a κ^3^*E,N,N′*-chloridotetryl ligand that arises from the insertion of the tetrylene E atom into an M–Cl bond and the coordination of an amidinate N atom and its closest quinolyl N atom to the metal center. Finally, the binuclear ruthenium(0) and cobalt(0) complexes [Ru_2_{μ_E_-κ^3^*E,N,N*′**-E(hmds)(bqfam)}(CO)_6_] and [Co_2_{μ_E_-κ^3^*E,N,N*′**-E(hmds)(bqfam)}(μ-CO)(CO)_4_] (E = Ge, Sn) have a related κ^3^*E,N,N*′**-tetrylene ligand that bridges two metal atoms through the E atom. For the κ^3^*E,N,N′-*metal complexes, the quinolyl fragment not attached to the metal is pendant in all the germanium compounds but, for the tin derivatives, is attached to (in the Pd and Pt complexes) or may interact with (in the Ru_2_ and Co_2_ complexes) the tin atom.

## Abstract 2

Amidinatotetrylenes donor functionalized on both nitrogen atoms have been prepared for the first time from *N,N′-bis*(quinol-8-yl)formamidine and Ge{N(SiMe_3_)_2_}_2_ or SnCl{N(SiMe_3_)_2_}. The resulting germylene and stannylene, decorated with two quinol-8-yl arms on both sides of the amidinato fragment, have led to metal complexes equipped with an unexpected pincer κ^3^*E,N,N′*-ligand configuration (E = Ge, Sn). Curiously, one of the two quinolyl fragments is pendant for the germanium compounds, but interacting with the tetrel atom for the tin derivatives.

## 1 Introduction

Heavier carbene analogues (silylenes, germylenes, stannylenes, and plumbylenes), also known as heavier tetrylenes (HTs),^1^ are increasingly utilized as ligands in coordination chemistry and homogeneous catalysis.^2,3^ Among the currently known HTs, those stabilized by amidinate fragments (amidinatotetrylenes, ATs) are playing a predominant role, since many of their metal complexes,^4,5^ more frequently those fitted with polydentate-AT variants,^5^ have shown remarkable activities in a wide variety of catalytic transformations.

**Figure 1:**
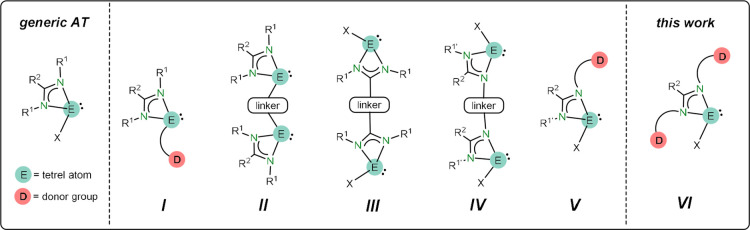
Types of currently known metal-free potentially polydentate ATs.

In this regard, the metal complexes that are equipped with polydentate ATs have been generally^6^ prepared from metal-free ATs (generically E(R^1^NC(R^2^)NR^1^)X; E = heavier tetrel atom, X = anionic group; see Figure 1) functionalized with at least one additional donor group (D). Figure 1 shows the currently known types of metal-free potentially polydentate ATs,^7−34^ which formally result from (a) attaching a donor fragment to the E atom (X position; type **I**),^7−17^ (b) connecting with a linker, which can also have additional donor or prone to undergo metalation groups, two ATs through their E atoms (X position; type **II**),^18−28^ through the amidinate central C atoms (R^2^ position; type **III**)^29−32^ or through one of the two amidinate N atoms (R^1^ position; type **IV**),^31^ and (c) attaching a donor fragment to one of the N atoms (R^1^ position; type **V**).^33,34^ By far, types **I** and **II**, whose syntheses normally imply an easy Cl replacement with an appropriate lithiated group on well-known chlorido-ATs,^35^ are the most explored donor-functionalized ATs, having led to a great variety of metal complexes fitted with κ^2^*E*,*D*-,^36−39^ κ^2^*E*,*E*-,^40,41^ κ^3^*E*,*D*,*E*-,^42^ κ^3^*E*,*C*,*E*-^43^ ligands, many of them with catalytic applications.^5^ On the other hand, the functionalization pathways that lead to types **III**, **IV**, and **V**, which imply modifications on the amidinate skeleton before the AT synthesis, have been comparatively much less studied. In fact, only a few of these systems, particularly of type **V**, have been involved (as ligands) in coordination chemistry. They have so far led to complexes featuring κ^2^*Ge,P*-,^33^ κ^3^*E,N,P*-,^34^ and κ^4^*E,N,P,P-*^33,34^ ligands (E = Ge, Sn).

**Figure 2:**
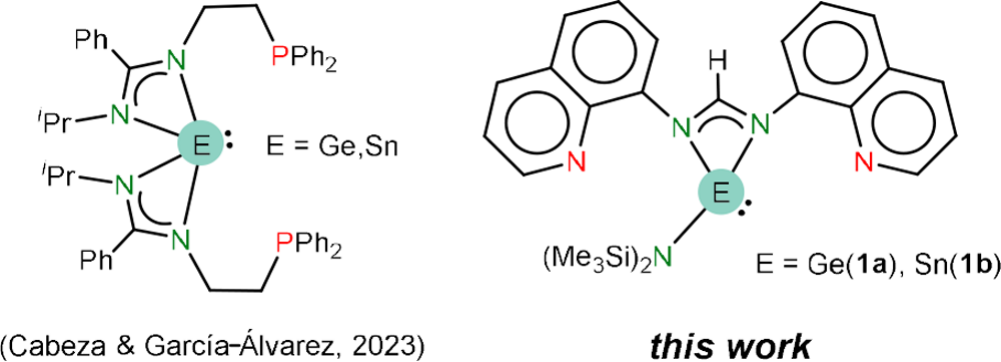
Currently known, including this work, potentially tridentate ligands featuring an AT fragment in the central position.

This work, prompted by the current lack of studies on the coordination chemistry of polydentate-ATs nondirectly donor functionalized on the tetrel atom (types **III**–**V**), adds an unexplored configuration to the field, describing the synthesis, characterization, and some coordination chemistry of E(hmds)(bqfam) (E = Ge (**1a**), Sn (**1b**); see Figure 2, right). These compounds are, as far as we are aware, the first *N,N*′**-bis(donor-functionalized)ATs. Germylene **1a** and stannylene **1b** are potentially tridentate ligands featuring an AT in the central position. Such a type of tridentate ligands has only been recently reported by our group for the PEP bis(amidinato)tetrylenes E(bzamP)_2_ (E = Ge, Sn; HbzamP = *N*-*iso*propyl-*N*′**-diphenylphosphanylethyl)benzamidine; see Figure 2, left),^34^ which are inscribed in the also rare type **V** category.

## 2 Results and Discussion

**Scheme 1:**
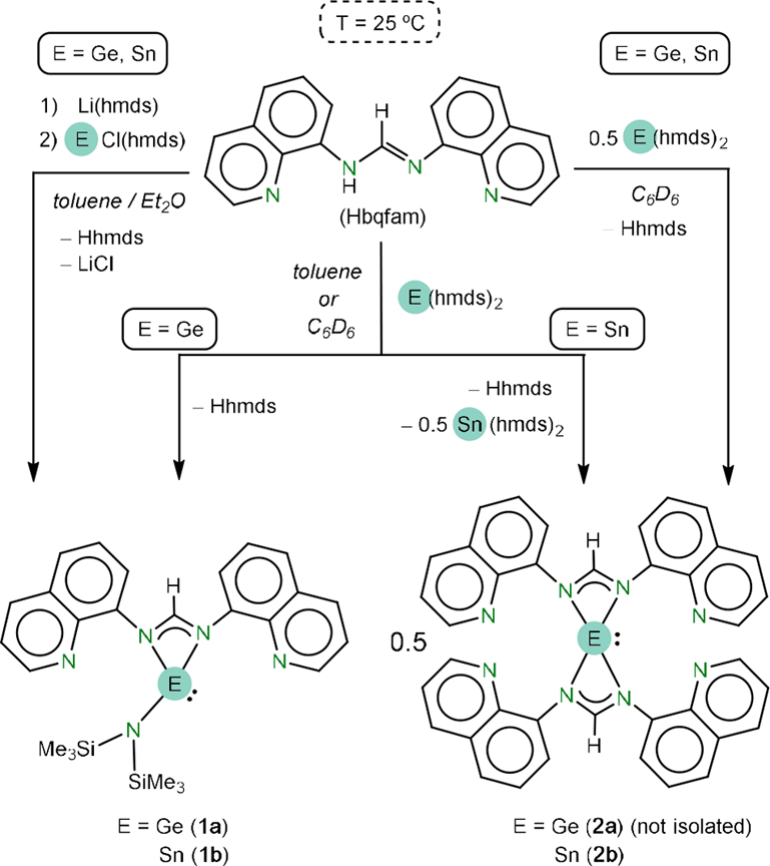
Reactions Leading to Compounds **1a**, **1b**, **2a**, and **2b**

The reactions of Hbqfam^44^ with E(hmds)_2_ (E= Ge, Sn) in a 1:1 ratio at room temperature led to different results depending on the nature of the E atom (Scheme 1, central pathway). For E= Ge, the deprotonation of the NH of Hbqfam by one of the hmds groups of Ge(hmds)_2_ led to Ge(hmds)(bqfam) (**1a**) (94% isolated yield). However, for E = Sn, the hmds group of Sn(hmds)(bqfam) (**1b**), which is presumably initially formed, can also deprotonate unreacted Hbqfam, leading to the bis(amidinato)stannylene Sn(bqfam)_2_ (**2b**) as the only observed AT reaction product (mixed with Hhmds and unreacted Sn(hmds)_2_). Stannylene **2b** was later rationally prepared (97% isolated yield) by a 2:1 reaction of Hbqfam and Sn(hmds)_2_ (Scheme 1, right pathway). These results show the higher basicity of the Sn-bonded hmds group (compared to that of the Ge analogue) and also indicate that the deprotonation of Hbqfam by Sn(hmds)(bqfam) (**1b**) is faster than that by Sn(hmds)_2_. Additionally, it has to be considered that tin, larger and more acidic than germanium, can accommodate easily two bqfam fragments. Heteroleptic stannylene **1b** was later successfully prepared (83% isolated yield) following a two steps procedure, first deprotonating Hbqfam with Li(hmds) followed by a reaction with chloridostannylene SnCl(hmds) (Scheme 1, left pathway). This method could also be used for the preparation of **1a**, albeit in lower yield than that attained using the reaction of Hbqfam with Ge(hmds)_2_ (77% vs 94% for the latter method). Aiming at synthesizing a germanium analogue of **2b**, namely, Ge(bqfam)_2_ (**2a**), a 2:1 reaction of Hbqfam and Ge(hmds)_2_ was carried out, leading to the formation of the expected product as major species, but it could not be satisfactorily isolated in a pure form (Scheme 1, right pathway).

The ^1^H NMR spectra of **1a** and **1b** in C_6_D_6_ are very similar, showing only six quinoline signals (each one integrating for 2 H) in addition to a highly deshielded signal corresponding to the central H atom of the formamidinate fragment (δ 9.61 (**1a**) and 9.95 (**1b**) ppm) and an intense (18 H) and sharp singlet corresponding to the hmds group (δ 0.37 (**1a**) and 0.36 (**1b**) ppm). These data (and also the corresponding ^13^C{^1^H} NMR spectra) are in agreement with the structure depicted for **1a** and **1b** in Scheme 1, in which the amidinate fragment is chelating the E atom (4-membered ENCN ring). Note that the great majority of the known metal-free ATs^2f,4,5^ show this chelating arrangement for the amidinate fragment (only bis(amidinato)tetrylenes have shown in certain cases that one of the two amidinato moieties is not chelating the E atom).^13^

**Figure 3:**
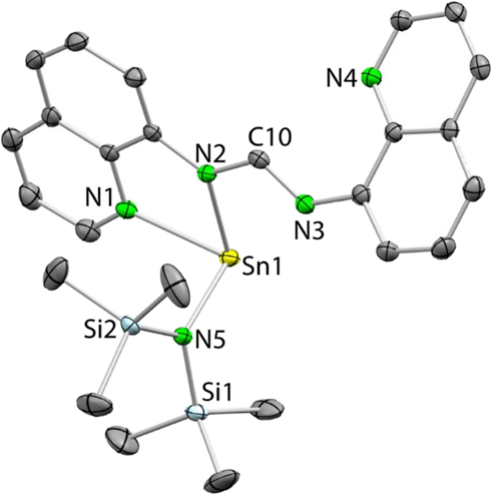
SCXRD molecular structure of **1b** [only one of the two positions in which one of the SiMe_3_ groups (Si1) is disordered is shown; 50% displacement ellipsoids; H atoms omitted for clarity]. Selected interatomic distances (Å) and angles (°): Sn1···N3 2.869(1), Sn1–N1 2.381(1), Sn1–N2 2.174(1), Sn1–N5 2.122(1) N2–C10 1.352(2), N3–C10 1.297(2); N3–C10–N2 116.4(1), N5–Sn1–N2 101.09(5), N5–Sn1–N1 93.05(5), N2–Sn1–N1 70.19(5).

The solid-state structure of stannylene **1b** was established by single-crystal X-ray diffraction (SCXRD) (Figure 3). Interestingly, in contrast with the suggestion of the nuclear magnetic resonance (NMR) spectra, in the solid state, the tin atom is not chelated by the amidinate fragment, but it is attached to an amidinate N atom (Sn1–N2 2.174(1) Å) and to its closest quinolyl N atom (Sn1–N1 2.381(1) Å), forming a 5-membered SnNCCN ring. The Sn–N bond distances, including Sn–N5 (2.122(1) Å), are similar to those previously found for related pyridyl–amide-stabilized hmds stannylenes.^45^ While the remaining quinoline group is clearly pendant, the remaining amidinato N atom (N3) is weakly interacting with the tin atom because the Sn1···N3 distance (2.869(1) Å) is clearly shorter than the sum of the vdW radii of both elements (3.72 Å).^46^ The different C10–N2 and C10–N3 distances (the latter is ca. 0.06 Å shorter) reflect the iminic character of the pendant amidinate N3 atom.

**Figure 4:**
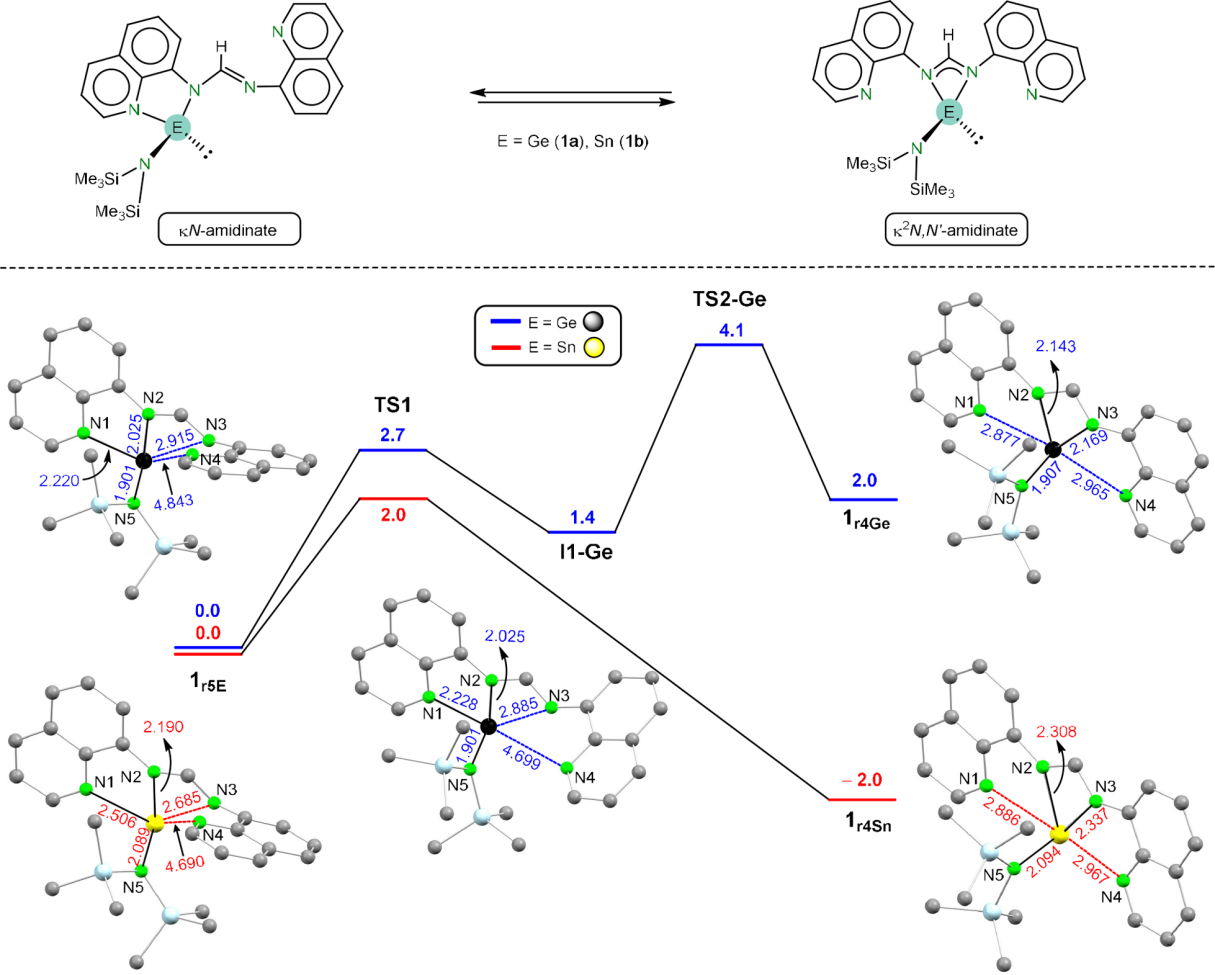
Dynamic behavior found for **1a** and **1b** in solution (top) and DFT-calculated (wB97xd/SDD_(Ge,Sn)_/cc-pVDZ) energy profile for the κ*N*-amidinate (**1**_**r5E**_) to κ^2^*N,N′*-amidinate (**1**_**r4E**_) interconversion for E= Ge, Sn. For clarity, the optimized structures of the transition states (TS) are not shown here but are shown in Figure S17. Gibbs energies (CPCM-toluene) are given in kcal mol^–1^. Interatomic distances are given in Å.

The asymmetric solid-state structure of **1b** is not maintained in solution (according to its NMR spectra). Therefore, a fluxional process that equilibrates both quinoline fragments, possibly also operating for germylene **1a**, might be operating in solution (Figure 4, top). In order to gain further insights into these processes, we modeled by density functional theory (DFT) calculations (Figure 4, bottom) the oscillation of the E atom of **1a** and **1b** from being attached to both amidinate N atoms (**1**_**r4E**_) to being chelated by an amidinate N atom and its closest quinolyl N atom (**1**_**r5E**_). For both tetrels, the computed Gibbs energy differences between the κ*N*-amidinate **1**_**r5E**_ and the κ^2^*N,N*′**-amidinate **1**_**r4E**_ are so small (≈ 2 kcal mol^–1^) that can be considered unsignificant (DFT energy calculations are affected by the used functional and atomic basis sets).^47^ The interconversion of **1**_**r5E**_ to **1**_**r4E**_ (Figure 4) is an elementary process for E = Sn but has two steps for E = Ge (through intermediate **I1**–**Ge**). Regarding the stannylene, the κ*N*-amidinate **1**_**r5Sn**_ (Sn1–N1 2.506, Sn1–N2 2.190 Å) evolves to **1**_**r4Sn**_ via an easily accessible transition state (**TS1**) in a process that implies, in addition to the κ^2^*N,N*′**-amidinate chelation, the rotation of the pendant quinolyl group to render a quasi-planar macrocycle where the amidinate and quinolyl N atoms are bonded (Sn1–N2 2.308, Sn1–N3 2.337 Å) or interacting (Sn1–N1 2.886, Sn1–N4 2.967 Å), respectively, with the tin atom. For the germylene, the **1**_**r5Ge**_ to **1**_**r4Ge**_ interconversion is analogous to that of the stannylene, but an intermediate (**I1**–**Ge**) was found in this case. Interestingly, the Ge–N distances involving the quinolyl N atoms of **1**_**r4Ge**_ are in the same range (Ge1–N1 = 2.877, Ge1–N4 = 2.965 Å) as those corresponding to **1**_**r4Sn**_, indicating that, possibly reflecting the larger acidity and higher tendency of tin to attain larger coordination numbers, these weak E–_N(quinolyl)_ interactions on **1**_**r4E**_ are stronger for tin. This fact is possibly related with **1**_**r4E**_ being, differently from the germylene, the most stable configuration for the stannylene. For E= Ge, the process has also a very low energy barrier (Δ*G*_(TS2-Ge)_ = 4.1 (E = Ge) kcal mol^–1^), which is in agreement with a variable temperature ^1^H NMR study carried out with **1a** (see Figure S2), which showed that while the Ge–N_(hmds)_ bond rotation is impeded at very low temperatures (the sharp singlet observed at room temperature for the two SiMe_3_ groups of hmds splits into too broad resonances), only one set of signals is observed for the two quinolyl fragments all the way down to −90 °C.

The structure of the bis(amidinato)stannylene Sn(bqfam)_2_ (**2b**) could not be unambiguously determined by SCXRD; however, its NMR data (^1^H and ^13^C in CD_2_Cl_2_) indicate that in solution, the four quinolyl fragments are equivalent. These data are in agreement with the structure depicted for **2b** in Scheme 1, where both amidinate fragments are chelating the Sn atom, however, considering the fluxional processes described above for **1a** and **1b** and having in mind the known tendency of bis(amidinato)-ATs to exhibit one amidinate fragment not chelating the E atom, many different isomeric structures, quickly exchanging in solution, are possible. Although the bis(amidinato)germylene Ge(bqfam)_2_ (**2a**) could not be isolated in a pure form, it is possibly the major product of the 2:1 reaction of Hbqfam and Ge(hmds)_2_ because the ^1^H NMR spectrum of the crude reaction outcome (see Figure S4) shows, in addition to the signals of other unidentified minor species, a set of signals very similar to that observed for stannylene **2b**.

**Scheme 2:**
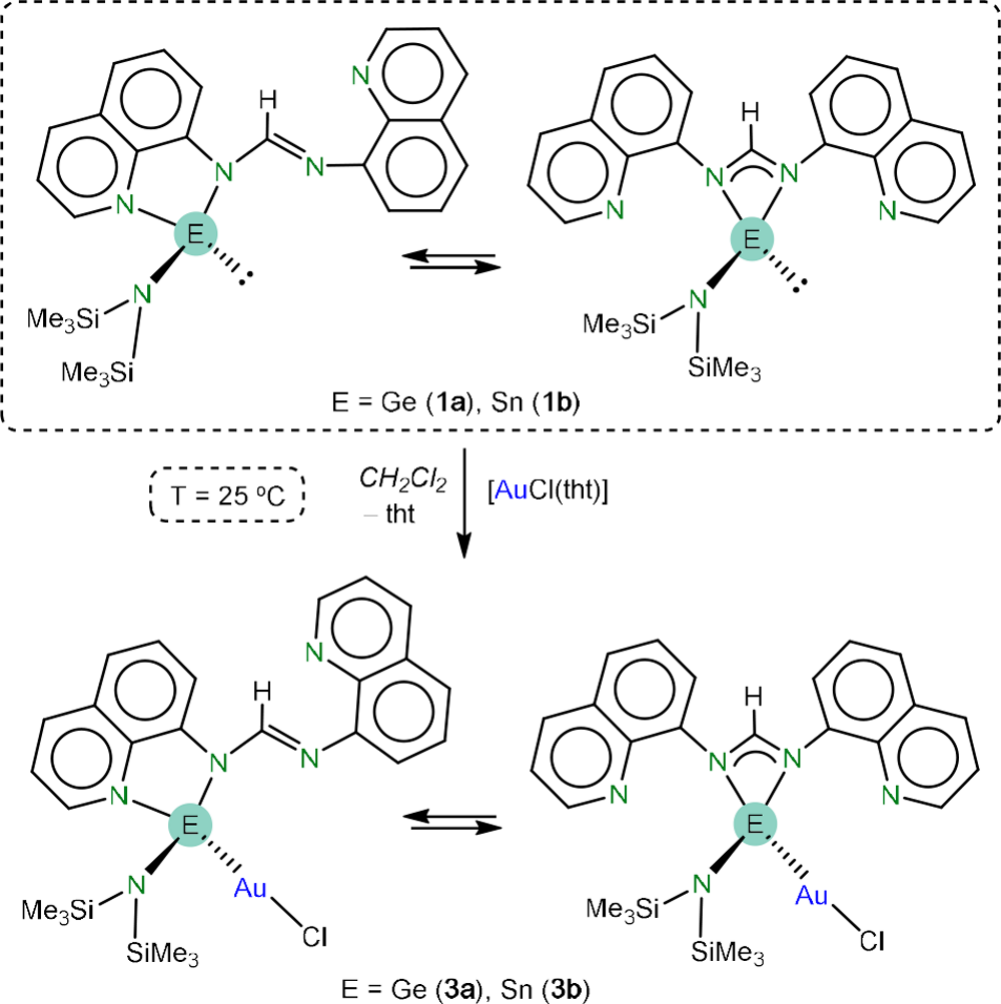
Syntheses of Compounds **3a** and **3b** and Their Dynamic Behavior in Solution

Considering that **1a** and **1b** represent a novel type of donor-functionalized ATs, we thought studying their reactivity toward transition metal complexes would be of interest. Their room temperature reactions with [AuCl(tht)] (tht = tetrahydrothiophene) in CH_2_Cl_2_ quickly led, upon tht replacement, to [AuCl{κ^1^*E*-E(hmds)(bqfam)} (E = Ge (**3a**), Sn (**3b**); Scheme 2) as the major reaction products (NMR analysis of crude reaction outcomes). Both complexes decomposed after standing in solution for long periods, with the formation of dark insoluble solids. Reasonably pure samples were obtained upon cooling to −20 °C saturated hexane/CH_2_Cl_2_ solutions of the complexes, which were isolated in yields of 84% (**3a**) and 46% (**3b**) as red and yellow crystals, respectively.

**Figure 5:**
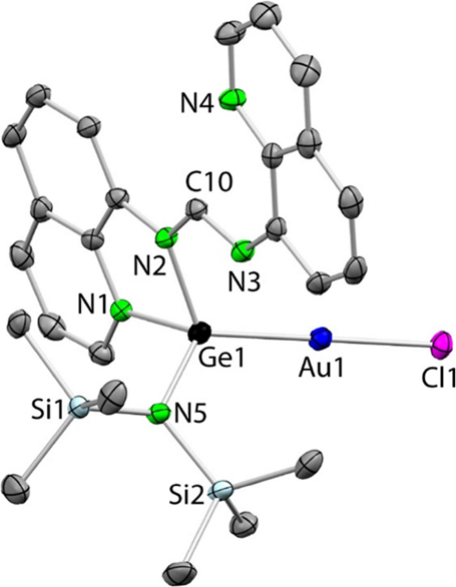
SCXRD molecular structure of **3a** (30% displacement ellipsoids; H atoms omitted for clarity). Selected interatomic distances (Å) and angles (deg): Au1–Ge1 2.3280(8), Au1–Cl1 2.306(2), Ge1···N3 2.963(5), Ge1–N1 2.025(6), Ge1–N2 1.905(5), Ge1–N5 1.841(5); N2–C10 1.364(9), N3–C10 1.283(8); Cl1–Au1–Ge1 177.81(5), N3–C10–N2 118.0(6), N5–Ge1–N2 113.7(2), N5–Ge1–N1 102.9(2), N2–Ge1–N1 81.6(2), N5–Ge1–Au1 121.2(2), N2–Ge1–Au1 117.3(2), N1–Ge1–Au1 111.4(2).

The SCXRD structure of germylene derivative **3a** (Figure 5) shows a linear gold(I) complex (Cl1–Au1–Ge1 177.81(5)°) featuring a monodentate κ*Ge*-germylene. Regarding the ligand conformation, it strongly resembles that of the free stannylene **1b** (Figure 3), showing also: (i) that the tetrel atom is not chelated by the amidinate fragment but attached to a amidinate N atom (Ge1–N2 1.905(5) Å) and to its closest quinolyl N atom (Ge1–N1 2.025(6) Å), forming, in this case, a 5-membered GeNCCN ring, (ii) that the remaining amidinate and quinolyl N atoms (N3 and N4) are pendant (the Ge1···N3 distance of 2.963(5) Å is also clearly shorter than the sum of the vdW radii of both elements, which is 3.66 Å),^46^ and (iii) that the C10–N2 and C10–N3 distances (the latter is ca. 0.06 Å shorter than the former) also reflect the iminic character of the pendant amidinate N3 atom. The Au–Ge bond distance (2.3280(8) Å) is very similar to those reported for other crystallographically characterized gold complexes equipped with neutral tricoordinated germylenes.^48^

The NMR data of **3a** and **3b** in CD_2_Cl_2_ are very similar to each other and also to those described above for **1a** and **1b**. For example, their ^1^H NMR spectra, in addition to the signals of the formamidinate central H atom (δ 9.74 (**3a**) and 10.11 (**3b**) ppm) and the hmds group (δ 0.24 (**3a**) and 0.12 (**3b**) ppm), show equivalent quinoline groups even at −90 °C, as evidenced by a variable temperature ^1^H NMR study carried out with **3a** (Figure S7). Again, the asymmetric solid-state structure of **3a** is not maintained in solution. Therefore, a fluxional process, similar to that described for **1a** and **1b**, is possibly also operating for **3a** and, in extension, for the analogous stannylene complex **3b** (Scheme 2).

**Scheme 3:**
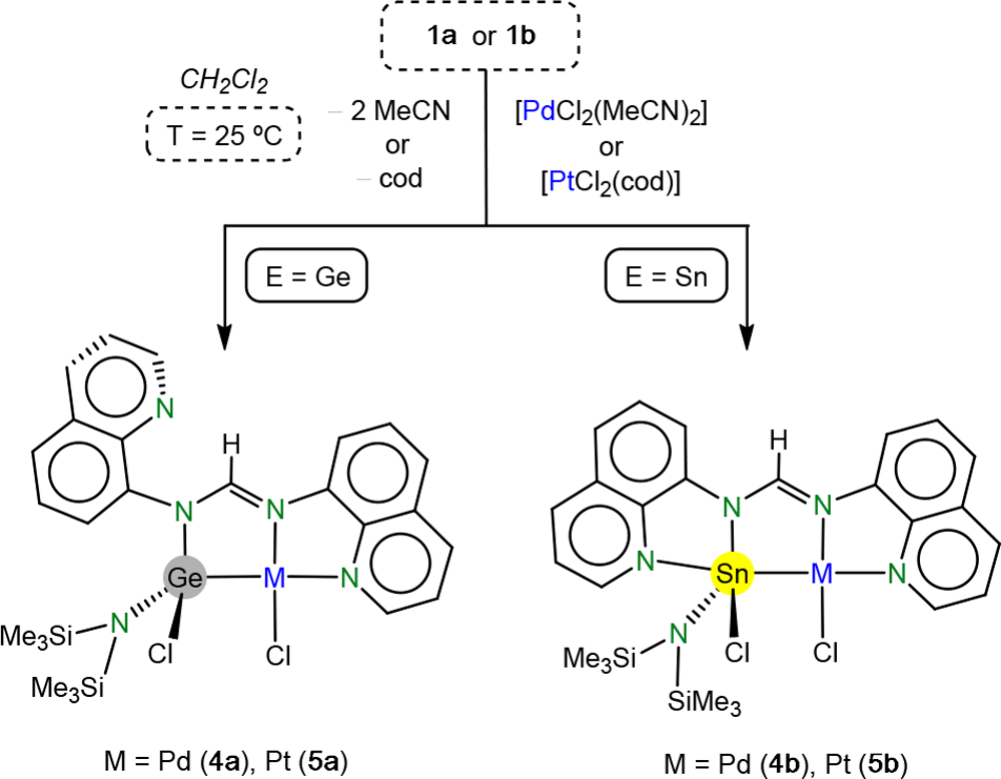
Syntheses of Compounds **4a**, **4b**, **5a**, and **5b**

The simple monodentate κ*E*-coordination found for **1a** and **1b** in gold complexes **3a** and **3b** changed drastically when other metals were used. In particular, the reactions of **1a** and **1b** with [PdCl_2_(MeCN)_2_] and [PtCl_2_(cod)] at room temperature led to the pincer-type derivatives [MCl{κ^3^*E,N,N*′**-ECl(hmds)(bqfam)}] (E = Ge: M = Pd (**4a**), Pt (**5a**); E = Sn: M = Pd (**4b**), Pt (**5b**)), which were isolated in moderate (46% for **5b**) to excellent yields (>84%) (Scheme 3).

**Figure 6:**
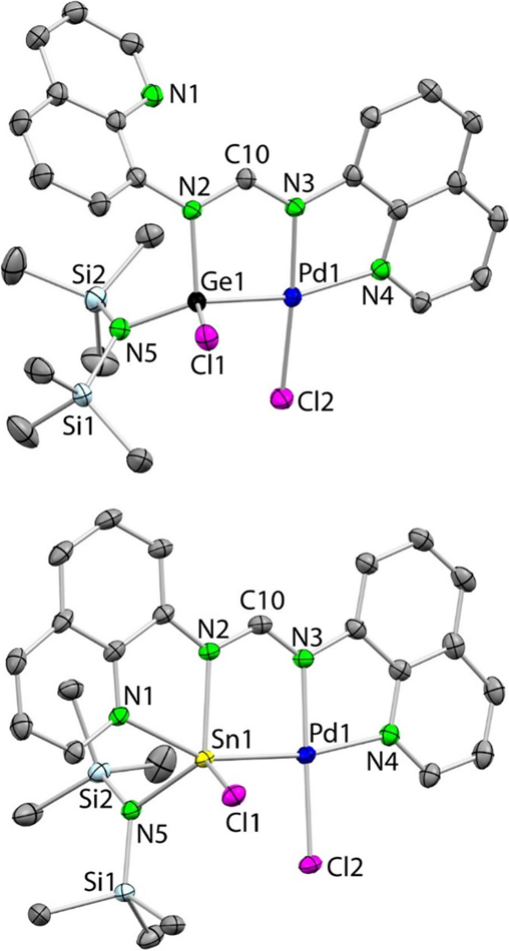
SCXRD molecular structures of **4a** (top) and **4b** (bottom) (30% displacement ellipsoids, H atoms omitted for clarity). Selected interatomic distances (Å) and angles (deg): **4a**: Pd1–Ge1 2.2839(5), Pd1–Cl2 2.298(1), Pd1–N3 2.004(3), Pd1–N4 2.106(3), Ge1–N2 1.971(3), Ge1–N5 1.820(4), Ge1–Cl1 2.211(1), N2–C10 1.339(5), N3–C10 1.320(5); N3–Pd1–N4 80.8(1), N3–Pd1–Ge1 84.78(9), N4–Pd1–Ge1 165.5(1), N3–Pd1–Cl2 176.1(1), N4–Pd1–Cl2 101.9(1), Ge1–Pd1–Cl2 92.56(3), N3–C10–N2 121.6(4), N5–Ge1–N2 109.3(1), N5–Ge1–Cl1 106.1(1), N2–Ge1–Cl1 98.5(1), N5 Ge1 Pd1 129.9(1), N2–Ge1–Pd1 95.8(1), Cl1–Ge1–Pd1 112.39(3). **4b**: Pd1–Sn1 2.4936(3), Pd1–Cl2 2.2967(9), Pd1–N3 2.016(3), Pd1–N4 2.124(3), Sn1–N1 2.314(3), Sn1–N2 2.248(3), Sn1–N5 2.107(3), Sn1–Cl1 2.431(1), N2–C10 1.336(5), N3–C10 1.305(5); N3–Pd1–N4 80.8(1), N3–Pd1–Sn1 89.64(9), N4–Pd1–Sn1 170.39(9), N3–Pd–Cl2 179.1(1), N4–Pd1–Cl2 98.82(9), Sn1–Pd1–Cl2 92.72(3), N3–C10–N2 122.4(3), N5–Sn1–N2 122.4(1), N5–Sn1–N1 85.6(1), N2–Sn1–N1 69.9(1), N5–Sn1–Cl1 116.40(9), N2–Sn1–Cl1 108.55(9), N1–Sn1–Cl1 81.76(9), N5 Sn1 Pd1 112.99(9), N2–Sn1–Pd1 85.44(8), N1–Sn1–Pd1 155.34(8), Cl1–Sn1–Pd1 105.97(3).

Figure 6 shows the SCXRD molecular structures of **4a** and **4b**. The structure of platinum complex **5a** was also established by SCXRD and is analogous to that of **4a** (see Figure S18). In addition to a roughly square planar metal coordination, the structures show a tridentate κ^3^*E,N,N*′**-chloridotetryl ligand that arises from the insertion of the tetrylene E atom into an M–Cl bond and the additional coordination of an amidinate N atom (N3) and its closest quinolyl N atom (N4) to the metal center. Curiously, the quinolyl fragment not attached to the metal is pendant in the germanium complex **4a** but attached to tin in **4b** (Sn1–N1 2.314(1) Å; note that the Sn–N_(quinolyl)_ distance in the free ligand **1b** is 2.381(3) Å). This difference (tetracoordinate germanium *vs* pentacoordinate tin) can be again attributed to the greater acidity of tin and its higher tendency to attain higher coordination numbers compared to those of germanium. The two quinolyl fragments are in an approximate *gauche* disposition in **4a** (the dihedral angle between the planes defined by the quinolyl planar rings is 58.31°) and in a perfect *syn* arrangement for **4b**, in such a way that the bqfam fragment is engaged in forming three fused roughly coplanar 5-membered rings. A similar μ–κ^4^*N*_4_-arrangement has been previously found for bqfam (or related *N*,*N*′-donor-functionalized amidinate ligands) in homometallic binuclear complexes of zinc, copper, palladium, platinum, etc.^49^ Within the amidinate fragment, the C10–N3 bond distances are only 0.02–0.03 Å shorter than the C10–N2 ones for both complexes, reflecting a higher degree of delocalization of the N=C double bond than that observed for the SCRXD characterized free ligand **1b** and gold complex **3a**, which feature pendant iminic N atoms.

The NMR data of the analogous germyl complexes **4a** and **5a** in CD_2_Cl_2_ are very similar to each other and, differently to what was observed for the free ligands **1a** and **1b** and the gold complexes **3a** and **3b**, they show inequivalent quinoline groups, in agreement with their molecular structures. For example, their ^13^C{^1^H} NMR spectra show, in addition to the formamidinate central CH group (δ 157.1 (**4a**) and 157.8 (**5a**) ppm) and the hmds group (δ 5.3 (**4a**) and 5.4 (**5a**) ppm), 18 different quinoline signals. The NMR data of the stannyl complexes **4b** and **5b** are also very similar to each other, therefore indicating that both compounds are structurally analogous and show, as expected, inequivalent quinoline groups. Curiously, different from the Ge-quinolyl-pendant compounds **4a** and **5a**, the Sn-quinolyl-attached derivatives **4b** and **5b** showed very low solubility in dichloromethane (see experimental section), also rendering diluted solutions even in THF-*d*_8_. This might explain the higher degree of hydrolysis (Hhmds detection) observed for the tin complexes in their NMR spectra.

**Scheme 4:**
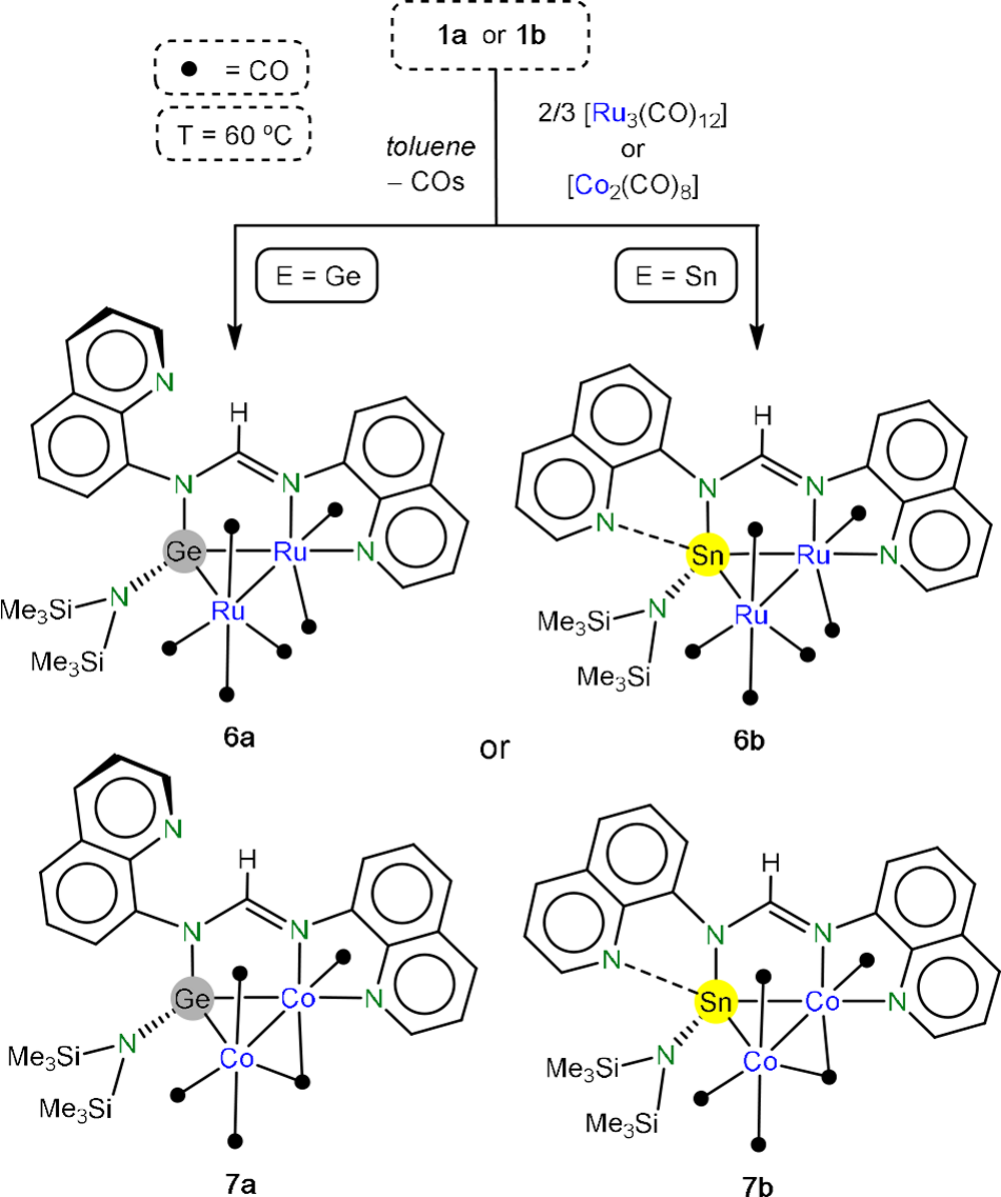
Syntheses of Compounds **6a**, **6b**, **7a**, and **7b**

Finally, the coordination chemistry study was extended to polynuclear complexes. The reactions of **1a,b** with 0.66 equiv of [Ru_3_(CO)_12_] or 1 equiv of [Co_2_(CO)_8_] at 60 °C led to the bimetallic derivatives [M_2_{μ_E_-κ^3^*E,N,N*′**-E(hmds)(bqfam)}(μ-CO)_x_(CO)_y_] (M = Ru; x = 0; y = 6: E = Ge (**6a**), Sn (**6b**). M = Co; x = 1; y = 4: E = Ge (**7a**), Sn (**7b**)), which were isolated in very good yields (80–90%) (Scheme 4). Note that the reactions of **1a,b** with [Ru_3_(CO)_12_] in a 1:1 ratio led to the same products, leaving some unreacted [Ru_3_(CO)_12_]. All complexes feature a tetrylene-bridging ligand that is additionally coordinated to one of the metals by one amidinate N atom and its closest quinolyl N atom, forming a μ_E_-κ^3^*E,N,N*′**-ligand, being the quinolyl fragment not attached to the metal, pendant for the germanium complexes **6a** and **7a**, and possibly weakly interacting with the tin atom for **6b** and **7b**. The coordination spheres of the two metals are completed by carbonyl ligands (six terminal for the ruthenium derivatives and four terminal and one bridging for the cobalt complexes).

**Figure 7:**
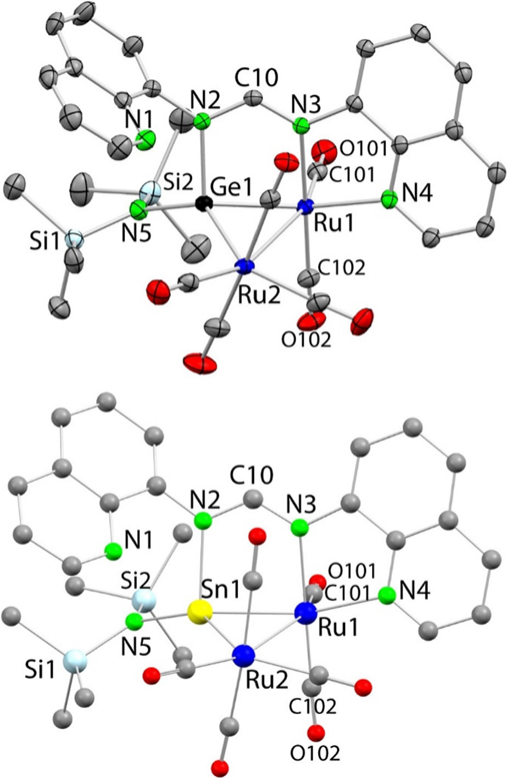
SCXRD molecular structure of **6a** (top; 30% displacement ellipsoids) and the DFT-optimized structure of **6b** (bottom). H atoms been omitted for clarity. Selected interatomic distances (Å) and angles (deg): **6a**: Ru1–Ge1 2.3898(3), Ru1–Ru2 2.9628(3), Ru1–N3 2.109(2), Ru1–N4 2.176(2), Ge1–N2 1.992(2), Ge1···N1 3.600(2), Ge1–N5 1.862(2), Ge1–Ru2 2.5063(3), N2–C10 1.330(3), N3–C10 1.320(3); N3–C10–N2 121.3(2), N5–Ge1–N2 102.6(1) N5–Ge1–Ru2 132.02(7), N2–Ge1–Ru2 111.38(6), N5–Ge1–Ru1 135.15(7), N2–Ge1–Ru1 95.91(6), Ru1–Ge1–Ru2 74.43(1) **6b**: Ru1–Sn1 2.560, Ru1–Ru2 3.130, Ru1–N3 2.152, Ru1–N4 2.202, Sn1–N2 2.209, Sn1···N1 3.044, Sn1–N5 2.024, Sn1–Ru2 2.662, N2–C10 1.324, N3–C10 1.320; N3–C10–N2 123.11, N5–Sn1–N2 100.65, N5–Sn1–Ru2 140.28, N2–Sn1–Ru2 109.14, N5Sn1Ru1 133.47, N2–Sn1–Ru1 89.34, Ru1–Sn1–Ru2 73.63.

The structures of these bimetallic complexes are proposed based on the following: (i) the molecular structure of the Ru_2_Ge derivative **6a** could be unambiguously determined by SCXRD (Figure 7, top), showing the aforementioned ligand coordination and a pendant quinolyl fragment, (ii) the DFT-optimized structure of the Ru_2_Sn complex **6b** (Figure 7, bottom), which is analogous to that of **6a**, shows a much shorter E···N_(quinolyl)_ distance, (iii) the pincer-type κ^3^*E,N,N*′**-ligand coordination mode exhibited by the ligands is essentially identical to that observed in the tetryl-palladium and -platinum complexes **4a**,**b** and **5a**,**b**, which also feature tetra- and penta-coordinated germanium and tin atoms, respectively, (iv) the ring opening of the EN_2_C four-membered ring of nondonor-functionalized amidinatogermylenes to produce related M_2_Ge (*M* = Ru, Co) carbonyl complexes has been previously described by our group.^50,51^ In particular, the compounds [Ru_2_{μ_Ge_-κ^2^*Ge,N*-GeX(^*i*^PrNC(Ph)N^*i*^Pr)}(CO)_7_] (X = hmds, ^*t*^Bu)^50^ and [Co_2_{μ_Ge_-κ^2^*Ge,N*-Ge(hmds)(^*i*^PrNC(Ph)^*i*^Pr)}(μ-CO)(CO)_5_]^51^ could be prepared in reactions of the corresponding germylenes with [Ru_3_(CO)_12_] or [Co_2_(CO)_8_], which differ with **6a**,**b** and **7a**,**b** in the number of carbonyl ligands (they have one more), since they are fitted with bidentate μ_Ge_-κ^2^*Ge,N-*ligands.

The geometrical parameters (Ru–Ru, Ru–Ge, Ge–N and Ru–N bond distances) of the SCXRD molecular structure of **6a** (Figure 7, top) are very similar to those previously reported for the aforementioned related Ru_2_Ge complexes.^50^ Within the amidinate fragment, the C–N bond distances differ by only 0.01 Å, reflecting a high degree of delocalization of the N=C bond. The geometrical parameters (Ru–Ru, Ru–Sn, Sn–N, and Ru–N bond distances) of the DFT-optimized structure of **6b** (Figure 7, bottom), compared to those of the SCXRD structure of **6a** (Figure 7, top), reflect the larger size of the tin atom (note that de DFT-optimized structure of **6a**, Figure S19, is closely related to its solid state structure). However, as previously mentioned, the distance between the N atom (N1) of the quinolyl fragment not attached to the metal and the tetrel atom is much shorter for tin (E···N1 = 3.600(2) (**6a**), 3.044 (**6b**) Å). While this Sn···N1 distance is clearly shorter than the sum of the vdW radii of both elements,^46^ indicating the existence of a weak interaction, it is much larger than the Sn–N_(quinolyl)_ bond distances found in the free ligand **1b** (2.381(1) Å) or in the stannyl-palladium complex **4b** (Sn1–N1 2.314(3) Å), where the quinolyl N atom is attached to tin.

The NMR data of all complexes are in agreement with the structures unambiguously established (**6a**) or proposed (**6b**, **7a**, and **7b**), since they show inequivalent quinoline groups (18 different quinoline signals can be found in the ^13^C{^1^H} NMR spectra). Differently to that observed for the tetryl-palladium and -platinum complexes, at room temperature, the E–N_(hmds)_ bond rotation is absent (for **6a** and **7a,b**) or impeded (for **6b**), since two different signals (broad for **6b**) are observed for the SiMe_3_ groups of hmds. This reflects the higher steric hindrance exerted by the M(CO)_4_ unit (*M* = Ru, Co) compared with that of the Cl atom attached to the tetrel atom in the corresponding complexes. A small (**6a**, **7a,b**) or high (**6b**) degree of hydrolysis (Hhmds detection), even in carefully dried CD_2_Cl_2_, was observed. Similarly to the tetryl-palladium and -platinum complexes, the tin derivatives **6b** and **7b** showed lower solubility than the germanium analogues **6a** and **7a**, which seems to be related to their main structural difference (short *vs* long E···N_(quinolyl)_ distances, respectively).

Emphasizing the novelty of the metal complexes described (M ≠ Au), a search in the Cambridge Crystallographic database^52^ showed that, while κ^3^*E,N*_2_-ligands (E = group-14 atom in no specific position within the ligand scaffold) are very well represented for E = Si,^53^ very few examples are known for the heavier group-14 elements; in particular, they are only known for E = Sn (tungsten complexes featuring κ^3^*N*_2_,Sn organotin-functionalized bis(pyrazol-1-yl)methane ligands).^54^

The coordination chemistry of bis(amidinato)stannylene Sn(bqfam)_2_ (**2b**) was not investigated in detail since an initial assessment resulted in discouraging results. For example, the reaction of [PdCl_2_(MeCN)_2_] with **2b** led to the formation of an intractable, very insoluble solid that could not be identified. The large amount of donor groups available in **2b**, which facilitates intermolecular interactions making the formation of different aggregates highly possible, may be behind this result.

## 3 Conclusions

In this work, we have prepared and characterized the compounds E(hmds)(bqfam) (E = Ge (**1a**), Sn (**1b**)), which are, as far as we are aware, the first *N,N′-*bis(donor)-functionalized amidinatotetrylenes, adding a novel type to this important family of ligands. The unique features of **1a** and **1b**, equipped with two quinol-8-yl arms on both sides of the amidinate fragment, allow the tetrel atom to easily oscillate in solution, in a fluxional process, from κ^2^*N,N*′**-amidinate to being chelated by an amidinate N atom and its closest quinolyl N atom, the latter being the preferred arrangement in the solid state (SCXRD structure of **1b**). This feature contrasts with the great majority of the metal-free ATs known,^2f,4,5^ which lack donor-functionalization on their N atoms and exhibit a closed κ^2^*N,N*′**-amidinate arrangement.

The coordination chemistry of **1a** and **1b** (we designed them looking for κ^3^*N,E,N*′**-ligands) reported in this work has shown that (i) a monodentate κ*E*-coordination is possible despite all the donor groups available (complexes **3a** and **3b**), (ii) a pincer tridentate κ^3^*E,N,N*′**-coordination, different from the expected κ^3^*N,E,N′-*one, is the preferred behavior (complexes **4a,b**–**7a,b**), as a consequence of the facile involvement of one of the amidinate N atoms in the coordination process, and, (iii) for the pincer metal complexes, one of the two quinolyl fragments is pendant for the germanium compounds but attached or interacting with the tetrel atom for the tin derivatives. This fact highlights the great versatility of HTs since a simple modification of the tetrel atom in isostructural ligands can lead to substantial structural changes.

## Supporting Information Available

No uncommon hazards are noted derived from the experimental work carried out; general procedures, experimental details, characterization data, and NMR spectra (PDF)
Atomic coordinates of DFT-optimized structures (XYZ)

## Supplementary Material

ic3c04135_si_001.pdf

ic3c04135_si_002.xyz
