# Supplementary material for: Amidinatotetrylenes Donor Functionalized on Both N Atoms: Structures and Coordination Chemistry
Source: Inorg Chem. 2024 Jan 30;63(6):3118–28. doi: 10.1021/acs.inorgchem.3c04135 (PMC10865366; doi:10.1021/acs.inorgchem.3c04135)
Supplement: Supplementary file 1 — ic3c04135_si_001.pdf [file ic3c04135_si_001.pdf]

SUPPORTING INFORMATION

Amidinatotetrylenes Donor-Functionalized on Both N Atoms: Structures and Coordination Chemistry

Christian Alonso,[†] *Javier A. Cabeza,**[,†] *Pablo García-Álvarez,**[,†] *Rubén García-Soriano*[†] *and Enrique Pérez-Carreño*[‡]

†Departamento de Química Orgánica e Inorgánica, Centro de Innovación en Química Avanzada ORFEO-CINQA, Universidad de Oviedo, E-33071 Oviedo, Spain

‡Departamento de Química Física y Analítica, Universidad de Oviedo, E-33071 Oviedo, Spain

General Procedures

All reactions and product manipulations were carried out under argon in a MBraun UNIlab Pro drybox or using Schlenk-vacuum line techniques. Unless otherwise stated, the reactions were carried out at room temperature. Solvents were dried over appropriate desiccating reagents and distilled under argon before being stored in the drybox in glass containers containing a bed of 4 Å molecular sieves. Compounds Ge(hmds)$_2$,[S1] Sn(hmds)$_2$,[S1] GeCl(hmds),[S1] SnCl(hmds),[S1] *N,N'*-bis(8-quinolyl)formamidine (Hbqfam),[S2] [PdCl$_2$(NCMe)$_2$],[S3] [PtCl$_2$(cod)][S4] and [AuCl(tht)][S5] were prepared following published procedures. All remaining reagents were purchased from commercial sources. All reagents and solvents were stored under argon in a drybox. All reaction products were vacuum-dried for several hours prior to being weighted and analysed. NMR spectra were run on Bruker NAV-400, AV-400 and AV-300 instruments using as standards the residual protic solvent resonance for ^{1}H [δ(C$_6$*H*D$_5$) 7.16 ppm; δ(C*H*DCl$_2$) 5.32 ppm; δ (C$_4$*H*D$_7$O) 3.58 ppm] and the solvent resonance for ^{13}C [δ(*C*$_6$D$_6$) 128.1 ppm; δ(*C*D$_2$Cl$_2$) 53.8 ppm; δ(*C*$_4$D$_8$O) 25.3 ppm]. FT-IR spectra were run on a Perkin Elmer Spectrum RX I spectrophotometer. Microanalyses were obtained with a Thermo-Finnigan FlashEA112 microanalyzer. High-resolution mass spectra (HRMS) were obtained with a Bruker Impact II mass spectrometer operating in the ESI-Q-TOF positive mode; data given refer to the most abundant isotopomer of the observed species with the greatest mass. CHN microanalyses were not obtained for **2a** and **3b** since, according to their NMR data, they were affected by unknown impurities that could not be removed. No uncommon hazards are noted derived from the experimental work carried out.

Experimental Details and Characterization Data

Ge(hmds)(bqfam) (1a): *Method (a):* Ge(hmds)$_2$ (787 mg, 2.0 mmol) was slowly added to a solution of Hbqfam (597 mg, 2.0 mmol) in toluene (5 mL) to give an orange suspension that was stirred for 3 h. All volatiles were removed *in vacuo* and the residue was washed with hexane (8 mL) and vacuum-dried to give **1a** as an orange solid (1.0 g, 94%). *Method (b):* Li(hmds) (84 mg, 0.5 mmol) and Hbqfam (150 mg, 0.5 mmol) were dissolved in toluene/diethyl ether (7:1, 4 mL) to give a yellow suspension that was stirred for 2 h. GeCl(hmds) (134 mg, 0.5 mmol) was then added and the resulting reddish orange suspension was stirred overnight. All volatiles were removed *in vacuo* and the resulting solid was extracted with toluene (3 × 4 mL). The combined extracts were filtered through a glass-fiber filter that was washed with toluene (1 mL), adding the resulting washings to the combined filtered extracts, which were vacuum-evaporated. The resulting residue was washed with hexane (2 mL) and vacuum-dried to give **1a** as an orange solid (204 mg, 77% yield). Anal. (%) calcd. for $C_{25}H_{31}GeN_5Si_2$ (M = 530.35 amu): C, 56.62; H, 5.89; N, 13.21; found: C, 56.08; H, 5.72; N, 13.12. (+)-HRMS: *m/z* 564.1265. Calcd. for $C_{26}H_{36}GeN_5OSi_2$: *m/z* 564.1675 $[M + MeOH + H]^+$. ^{1}H NMR (C_6D_6, 300.1 MHz, 298 K): δ 9.61 (s, 1 H, NC*H*N), 8.41 (d, J = 6.0 Hz, 2 H), 7.67–7.37 (m, 4 H), 7.22 (t, J = 6.0 Hz, 2 H), 6.95 (d, J = 9.0 Hz, 2 H), 6.63 (dd, J = 6.0 and 3.0 Hz, 2 H), 0.37 (s, 18 H, 2 Si(C*H*$_3$)$_3$) ppm. ^{13}C{^{1}H} NMR (C_6D_6, 75.5 MHz, 298 K): δ154.6 (s, N*C*HN), 146.5 (s, 2 *C*s), 146.3 (s, 2 *C*Hs), 141.7 (s, 2 *C*s), 137.2 (s, 2 *C*Hs), 129.9 (s, 2 *C*s), 128.8 (s, 2 *C*Hs), 121.3 (s, 2 *C*Hs), 118.7 (s, 2 *C*Hs), 117.2 (s, 2 *C*Hs), 5.9 (s, 2 Si(*C*H$_3$)$_3$) ppm.

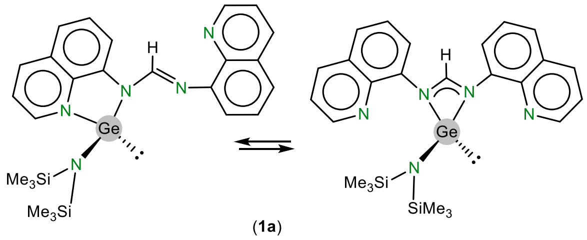

(**1a**)

Sn(hmds)(bqfam) (1b): Li(hmds) (84 mg, 0.5 mmol) and Hbqfam (150 mg, 0.5 mmol) were dissolved in toluene/Et$_2$O (7:1, 4 mL) to give a yellow suspension that was stirred for 2 h. SnCl(hmds) (158 mg, 0.5 mmol) was then added and the resulting bright-orange suspension was stirred overnight. Hexane (8 mL) was added and the suspension was filtered through a glass-fibre filter. The filtrate was vacuum-evaporated and the resulting residue was washed with hexane (2 × 5 mL) and vacuum-dried to give **1b** as an orange solid (239 mg, 83%).

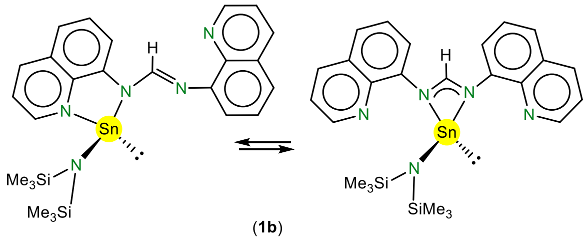

(**1b**)

Anal. (%) calcd. for $C_{25}H_{31}N_5Si_2Sn$ (M = 576.43 amu): C, 52.09; H, 5.42; N, 12.15; found: C, 43.98; H, 2.95; N, 10.49 (possibly affected by the air-sensitivity of the compound, this analysis provides the best values obtained to date). (+)-HRMS: The molecular ion (M^+) and/or its fragments could not be identified in the obtained spectra. ^1H NMR (C_6D_6, 400.1 MHz, 298 K): δ 9.95 (s, 1 H, NC*H*N), 8.53 (dd, *J* = 4.3 and 1.6 Hz, 2 H), 7.51 (dd, *J* = 8.2 and 1.6 Hz, 2 H), 7.36 (dd, *J* = 7.7 and 1.2 Hz, 2 H), 7.22 (t, *J* = 7.7 Hz, 2 H), 7.01 (dd, *J* = 8.2 and 1.2 Hz, 2 H), 6.71 (dd, *J* = 8.2 and 4.3 Hz, 2 H), 0.36 (s, 18 H, 2 Si(C*H*$_3$)$_3$) ppm. $^{13}C\{^1H\}$ NMR (C_6D_6, 75.5 MHz, 298 K): δ155.2 (s, N*C*HN), 147.6 (s, 2 *C*Hs), 144.3 (s, 2 *C*s), 142.7 (s, 2 *C*s), 137.0 (s, 2 *C*Hs), 130.1 (s, 2 *C*s), 121.4 (s, 2 *C*Hs), 119.7 (s, 2 *C*Hs), 116.0 (s, 2 *C*Hs), 6.5 (s, 2 Si(*C*H$_3$)$_3$) ppm. $^{119}Sn\{^1H\}$ (C_6D_6, 149.2 MHz, 298 K): not found.

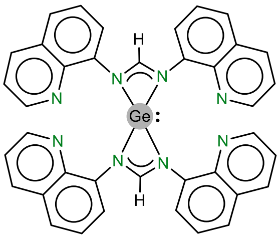

(**2a**)

Reaction of Hbqfam with Ge(hmds) in a 2:1 ratio; formation of Ge(bqfam)$_2$ (2a): Hbqfam (30 mg, 0.1 mmol) was dissolved in C_6D_6 (1 mL) and Ge(hmds)$_2$ (20 mg, 0.05 mmol) was slowly added to give a red suspension. After stirring for 15 min, an aliquot of the reaction crude was evaporated and analysed by ^1H NMR in CD_2Cl_2, showing the formation of **2a** as the major reaction product. All volatiles were removed *in vacuo* and the residue was washed with hexane (2 × 4 mL) and vacuum dried to give an orange solid (33 mg), which correspond to possibly **2a** as major compound, solvents and other unidentified species. Further washing with hexane did not allow the isolation of **2a** as a pure compound. ^1H NMR (CD_2Cl_2, 300.1 MHz, 298 K; signals attributed to **2a**): δ 9.28 (s, 4 H), 8.38 (s, 2 H, NC*H*N), 8.15 (d, *J* = 8.0 Hz, 4 H), 7.49–7.25 (m, 12 H), 7.14 (d, *J* = 6.6 Hz, 4 H) ppm.

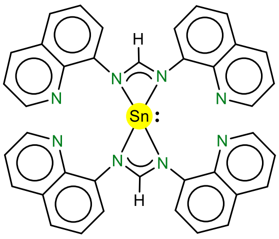

(**2b**)

Reactions of Hbqfam with Sn(hmds); synthesis of Sn(bqfam)$_2$ (2b): <u>*1:1 reaction:*</u> Sn(hmds)$_2$ (220 mg, 0.5 mmol) was dissolved in C_6D_6 (0.5 mL) and Hbqfam (141 mg, 0.5 mmol) was slowly added to give a dark orange suspension. After stirring for 3 h, an aliquot of the reacting solution was diluted with C_6D_6 and analyzed by ^1H NMR, showing the presence of **2b**, Hhmds and unreacted Sn(hmds)$_2$ as the three main products. All volatiles were removed in vacuo and the resulting dark orange solid was washed with hexane (4 × 2 mL) and then with hexane/toluene (1:1, 3 × 4 mL) and vacuum-dried to give **2b** as a red solid (152 mg, 85% respect to Hbqfam). <u>*2:1 reaction:*</u> Hbqfam (60 mg, 0.2 mmol) was dissolved in C_6D_6 (0.5

mL) and $Sn(hmds)_2$ (44 mg, 0.1 mmol) was slowly added to give a red suspension that was stirred for 15 minutes. All volatiles were removed in vacuo and the resulting residue was washed with hexane (4 mL) and vacuum-dried to give **2b** as a red solid (69 mg, 97 % yield). Anal. (%) calcd. for $C_{38}H_{26}N_8Sn$ (M = 713.38 amu): C, 63.99; H, 3.67; N, 15.71; found: C, 61.90; H, 3.80; N, 14.29. (possibly affected by the air-sensitivity of the compound, this analysis provides the best values obtained to date). (+)-HRMS: The molecular ion (M^+) and/or its fragments could not be identified in the obtained spectra. ^{1}H NMR (CD_2Cl_2, 300.1 MHz, 298 K): δ 9.45 (s, 4 H), 8.72 (s, 2 H, NC*H*N), 8.24 (d, *J* = 9.0 Hz, 4 H), 7.46 (s, 12 H), 7.05 (d, *J* = 3.0 Hz, 4 H) ppm. ^{13}C{^{1}H} NMR (CD_2Cl_2, 100.6 MHz, 298 K): δ 157.8 (s, N*C*HN), 149.8 (s, 4 *C*H), 148.5 (s, 4 *C*s), 143.0 (s, 4 *C*s), 137.2 (s, 4 *C*Hs), 129.8 (s, 4 *C*s), 127.8 (s, 4 *C*Hs), 121.4 (s, 4 *C*Hs), 120.2 (s, 4 *C*Hs), 117.0 (s, 4 *C*Hs) ppm. ^{119}Sn{^{1}H} NMR (CD_2Cl_2, 149.2 MHz, 298 K): not found.

[AuCl{κ^1*Ge*-Ge(hmds)(bqfam)}] (3a): Germylene **1a** (53 mg, 0.1 mmol) and [AuCl(tht)] (32 mg, 0.1 mmol) were simultaneously dissolved with CH_2Cl_2 (6 mL) to give a dark red solution that was filtered after 15 min of stirring through a glass-fiber filter. Addition of hexane (4 mL) and cooling at –20 °C overnight afforded a crop of red crystals. The crystals were separated by decantation, washed with hexane (1 mL) and vacuum-dried to give **3a** as red crystals (64 mg, 84 % yield). Anal. (%) calcd. for $C_{25}H_{31}AuClGeN_5Si_2$ (*M* = 762.75 amu): C, 39.37; H, 4.10; N, 9.18; found: C, 38.04; H, 4.01; N, 8.70 (possibly affected by the air-sensitivity of the compound, this analysis provides the best values obtained to date). (+)-HRMS: The molecular ion (M^+) and/or its fragments could not be identified in the obtained spectra. ^{1}H NMR (CD_2Cl_2, 400.1 MHz, 298 K): δ 9.74 (s, 1 H, NC*H*N), 8.85 (d, *J* = 4.5 Hz, 2 H), 8.42 (dd, *J* = 8.3 Hz, 1.1 Hz, 2 H), 7.71–7.48 (m, 8 H), 0.24 (s, 18 H, 2 Si(C*H*$_3$)$_3$) ppm. ^{13}C{^{1}H} NMR (CD_2Cl_2, 100.6 MHz, 298 K): δ 151.7 (s, N*C*HN), 147.0 (s, 2 *C*Hs), 142.0 (s, 2 *C*s), 139.6 (s, 2 *C*Hs), 139.3 (s, 2 *C*s), 129.8 (s, 2 *C*s), 129.3 (s, 2 *C*Hs), 122.5 (s, 2 *C*Hs), 120.8 (s, 2 *C*Hs), 117.8 (s, 2 *C*Hs), 5.6 (s, 2 Si(*C*H$_3$)$_3$) ppm.

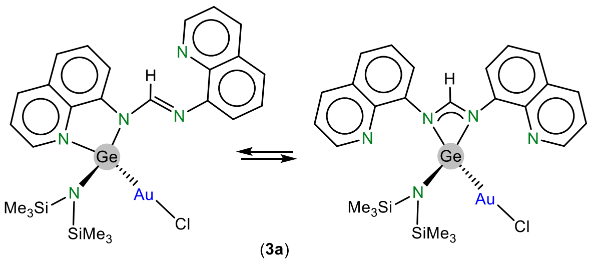

(3a)

[AuCl{κ^1*Sn*-Sn(hmds)(bqfam)}] (3b): Stannylene **1b** (17 mg, 0.03 mmol) and [AuCl(tht)] (10 mg, 0.03 mmol) were simultaneously dissolved in CH_2Cl_2 (3 mL) to give a dark red solution which was stirred for 15 min. Addition of hexane (1 mL) and cooling

at –20 °C overnight afforded a crop of yellow crystals. The crystals were separated by decantation, washed with hexane (0.5 mL) and vacuum-dried to give **3b** as yellow crystals (11 mg, 46 % yield). (+)-HRMS: The molecular ion

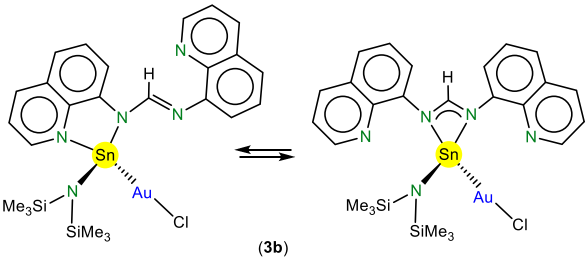

(**3b**)

(M^+) and/or its fragments could not be identified in the obtained spectra. ^{1}H NMR (CD_2Cl_2, 400.1 MHz, 298 K): δ 10.11 (s, 1 H, NC*H*N), 8.92 (d, *J* = 4.0 Hz, 2 H), 8.31 (d, *J* = 7.9 Hz, 2 H), 7.70–7.33 (m, 8 H), 0.12 (s, 18 H, 2 Si(C*H*$_3$)$_3$) ppm. ^{13}C{^{1}H} NMR (CD_2Cl_2, 100.6 MHz, 298 K): δ 151.6 (s, N*C*HN), 148.4 (s, 2 *C*Hs), 140.5 (s, 2 *C*s), 138.3 (s, 2 *C*Hs), 138.1 (s, 2 *C*s), 129.9 (s, 2 *C*s), 128.1 (s, 2 *C*Hs), 122.7 (s, 2 *C*Hs), 122.1 (s, 2 *C*Hs), 115.5 (s, 2 *C*Hs), 6.1 (s, 2 Si(*C*H$_3$)$_3$) ppm. ^{119}Sn{^{1}H} NMR: not found.

[PdCl{κ^3*Ge,N,N'*-GeCl(hmds)(bqfam)}] (4a): Germylene **1a** (26 mg, 0.05 mmol) was added to a solution of [$PdCl_2(NCMe)_2$] (13 mg, 0.05 mmol) in dichloromethane (1 mL) to give an ocher solution that was stirred for 15 min. All volatiles were removed *in vacuo*, affording a residue that was vacuum-dried to give **4a**

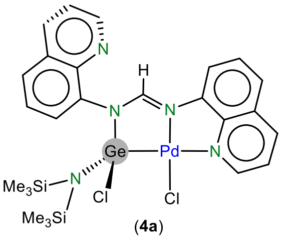

(**4a**)

as an ocher solid (35 mg, 99%). Anal. (%) calcd. for $C_{25}H_{31}Cl_2GeN_5PdSi_2$ (*M* = 707.67 amu): C, 42.43; H, 4.42; N, 9.90; found: C, 41.12; H, 4.14; N, 9.17 (possibly affected by the air-sensitivity of the compound, this analysis provides the best values obtained to date). (+)-HRMS: *m/z* 672.0032. Calcd. for $C_{25}H_{31}ClGeN_5PdSi_2$: *m/z* 672.0065 [*M* – Cl]$^+$. ^{1}H NMR (CD_2Cl_2, 300.1 MHz, 298 K): δ 9.04 (d, *J* = 3.0 Hz, 1 H), 8.95 (s, 1 H, NC*H*N), 8.88 (d, *J* = 6.0 Hz, 1 H), 8.40 (d, *J* = 9.0 Hz, 1 H), 8.28 (d, *J* = 9.0 Hz, 1 H), 8.18 (d, *J* = 9.0 Hz, 1 H), 7.80–7.42 (m, 7 H), 0.24 (s, 18 H, 2 Si(C*H*$_3$)$_3$) ppm. ^{13}C{^{1}H} NMR (CD_2Cl_2, 75.5 MHz, 298 K): δ 157.1 (s, N*C*HN), 150.6 (s, *C*H), 148.6 (s, *C*H), 145.9 (s, *C*), 145.0 (s, *C*), 143.3 (s, *C*), 139.1 (s, *C*H), 138.0 (s, *C*), 137.0 (s, *C*H), 130.8 (s, *C*), 129.8 (s, *C*), 128.4 (s, *C*H), 126.7 (s, *C*H), 126.5 (s, *C*H), 125.1 (s, *C*H), 123.6 (s, *C*H), 122.8 (s, *C*H), 122.1 (s, *C*H), 115.3 (s, *C*H), 5.3 (s, 2 Si(*C*H$_3$)$_3$) ppm.

[PdCl{κ^3*Sn,N,N'*-SnCl(hmds)(bqfam)}] (4b): Stannylene **1b** (30 mg, 0.05 mmol) and [$PdCl_2(NCMe)_2$] (13 mg, 0.05 mmol) were dissolved in dichloromethane (0.5 mL) to give an orange suspension that was stirred for 1 h. The supernatant liquid was decanted off and the precipitate was washed with

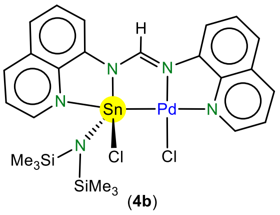

(**4b**)

dichloromethane (2 + 1 mL) and vacuum-dried to give **4b** as an orange solid (32 mg, 85%). Anal. (%) calcd. for $C_{25}H_{31}Cl_2N_5PdSi_2Sn$ (*M* = 753.75 amu): C, 39.84; H, 4.15; N, 9.29; found: C, 39.52; H, 3.83; N, 9.09. (+)-HRMS: *m/z* 931.0706. Calcd. for $C_{32}H_{44}Cl_2N_8NaOPdSi_2Sn$: *m/z* 931.05558 [M + 3 MeCN + MeOH + Na]$^+$. ^{1}H NMR (THF-d_8, 400.5 MHz, 298 K): δ 9.62 (d, *J* = 4.8 Hz, 1 H), 9.22 (d, *J* = 4.5 Hz, 1 H), 9.20 (s, 1 H, NC*H*N), 8.61 (d, *J* = 8.2 Hz, 1 H), 8.47 (d, *J* = 8.4 Hz, 1 H), 8.28 (d, *J* = 7.1 Hz, 1 H), 8.03 (d, *J* = 6.7 Hz, 1 H), 7.87 (dd, *J* = 8.0 and 4.8 Hz, 1 H), 7.72 (dd, *J* = 8.5 and 4.8 Hz, 1 H), 7.68–7.56 (m, 4 H), 0.26 (s, 18 H, 2 Si(C*H*$_3$)$_3$) ppm. ^{13}C{^{1}H} NMR (THF-d_8, 100.6 MHz, 298 K): δ149.8 (s, N*C*HN), 149.2 (s, *C*H), 148.9 (s, *C*H), 148.1 (s, *C*), 145.9 (s, *C*), 141.3 (s, *C*H), 139.2 (s, *C*H), 138.8 (s, *C*), 136.1 (s, *C*), 131.6 (s, *C*), 130.4 (s, *C*), 129.5 (s, *C*H), 128.8 (s, *C*H), 123.6 (s, *C*H), 123.5 (s, *C*H), 123.4 (s, *C*H), 121.6 (s, *C*H), 117.0 (s, *C*H), 115.7 (s, *C*H), 6.0 (s, 2 Si(*C*H$_3$)$_3$) ppm. ^{119}Sn{^{1}H} NMR (CD_2Cl_2, 149.2 MHz, 298 K): not found.

[PtCl{κ^3*Ge,N,N'*-GeCl(hmds)(bqfam)}] (5a): Germylene **1a** (53 mg, 0.1 mmol) was added to a suspension of [PtCl$_2$(cod)] (37 mg, 0.1 mmol) in benzene (1 mL) to give an orange suspension that was stirred for 15 min. All volatiles were removed *in vacuo*, leading to a yellow solid that was vacuum-dried to give **5a** (38 mg, 95%). Anal. (%) calcd. for $C_{25}H_{31}Cl_2GeN_5PtSi_2$ (*M* = 796.34 amu): C, 37.71; H, 3.92; N, 8.79; found: C 38.62; H, 3.67; N, 8.39 (possibly affected by the air-sensitivity of the compound, this analysis provides the best values obtained to date). (+)-HRMS: *m/z* 760.0651. Calcd. for $C_{25}H_{31}ClGeN_5PtSi_2$: *m/z* 760.0667 [*M* – Cl]$^+$. ^{1}H NMR (CD_2Cl_2, 300.1 MHz, 298 K): δ 9.26 (br s, 1 H), 8.91 (br s, 1 H), 8.82 (s, 1 H, NC*H*N), 8.50 (d, *J* = 9.0 Hz, 1 H), 8.33–8.18 (m, 2 H), 7.86–7.42 (m, 7 H), 0.27 (s, 18 H, 2 Si(C*H*$_3$)$_3$) ppm. ^{13}C{^{1}H} NMR (CD_2Cl_2, 75.5 MHz, 298 K): δ157.8 (s, N*C*HN), 150.5 (s, *C*H), 147.1 (s, *C*H), 146.6 (s, *C*), 145.3 (s, *C*), 143.1 (s, *C*), 139.3 (s, *C*H), 138.2 (s, *C*), 137.0 (s, *C*H), 130.9 (s, *C*), 129.9 (s, *C*), 128.7 (s, *C*H), 126.7 (s, *C*H), 126.3 (s, *C*H), 124.9 (s, *C*H), 123.9 (s, *C*H), 123.0 (s, *C*H), 122.0 (s, *C*H), 115.4 (s, *C*H), 5.4 (s, 2 Si(*C*H$_3$)$_3$) ppm.

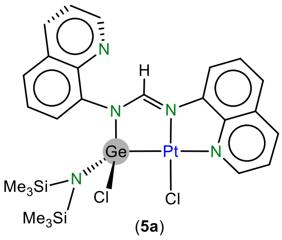
(**5a**)

[PtCl{κ^3*Sn,N,N'*-SnCl(hmds)(bqfam)}] (5b): Stannylene **1b** (35 mg, 0.06 mmol) and [PtCl$_2$(cod)] (22 mg, 0.06 mmol) were dissolved in dichloromethane (2 mL) to give an orange suspension that was stirred for 1 h. The supernatant liquid was decanted off and the precipitate was washed with

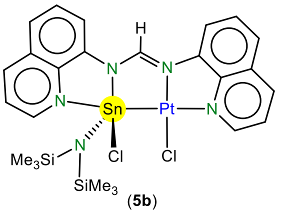
(**5b**)

dichloromethane (2 mL) and vacuum-dried to give **5b** as an orange solid (23 mg, 46 % yield). Anal. (%) calcd. for $C_{25}H_{31}Cl_2N_5PtSi_2Sn$ (*M* = 842.42 amu): C, 35.64; H, 3.71; N, 8.31; found: C, 35.69; H, 3.40; N, 7.96. (+)-HRMS: *m/z* 943.0637. Calcd. for $C_{30}H_{46}ClN_6O_3PtSi_2Sn$: *m/z* 943.15057 [M – Cl + MeCN + 3 MeOH]$^+$. ^{1}H NMR (THF-d_8, 400.5 MHz, 298 K): δ 9.77 (dd, *J* = 4.9 and 1.5 Hz, 1 H), 9.49 (dd, *J* = 4.8 and 1.4 Hz, 1 H), 8.78 (s, 1 H, NC*H*N), 8.60 (dd, *J* = 8.2 and 1.4 Hz, 1 H), 8.57 (dd, *J* = 8.4 and 1.4 Hz, 1 H), 8.27 (dd, *J* = 7.3 and 1.6 Hz, 1 H), 8.03 (dd, *J* = 7.4 and 1.3 Hz, 1 H), 7.84 (dd, *J* = 8.2 and 4.9 Hz, 1 H), 7.78 (dd, *J* = 8.4 and 4.9 Hz, 1 H), 7.66–7.56 (m, 4 H,), 0.29 (s, 18 H, 2 Si(C*H*$_3$)$_3$) ppm. ^{13}C{^{1}H} NMR (THF-d_8, 100.6 MHz, 298 K): δ 150.2 (s, N*C*HN), 149.3 (s, *C*H), 147.2 (s, *C*H), 146.3 (s, *C*), 145.4 (s, *C*), 141.3 (s, *C*H), 139.5 (s, *C*H), 139.0 (s, *C*), 136.5 (s, *C*), 131.8 (s, *C*), 130.6 (s, *C*), 129.6 (s, *C*H), 129.1 (s, *C*H), 123.7 (s, *C*H), 123.6 (s, *C*H), 123.1 (s, *C*H), 121.6 (s, *C*H), 116.9 (s, *C*H), 116.0 (s, *C*H), 5.9 (s, 2 Si(*C*H$_3$)$_3$) ppm.

[Ru$_2${μ$_{Ge}$-κ^3*Ge,N,N'*-Ge(hmds)(bqfam)}(CO)$_6$] (6a): [$Ru_3(CO)_{12}$] (42 mg, 0.066 mmol) was added to a solution of germylene **1a** (53 mg, 0.1 mmol) in toluene (5 mL) in a J. Young ampoule to give a dark red suspension, which was heated at 60 °C for 1.5 h (the ampoule was connected open to a Schlenk-vacuum line). The resultant suspension was filtered with a glass-fiber filter and the filter was washed with toluene (2 mL). All filtrates were collected and evaporated *in vacuo* to give **5a** as a dark red solid (73 mg, 81%). Anal. (%) calcd. for $C_{31}H_{31}GeN_5O_6Ru_2Si_2$ (*M* = 900.54 amu): C, 41.34; H, 3.47; N, 7.78; found: C, 42.16; H, 3.13; N, 8.04 (possibly affected by the air-sensitivity of the compound, this analysis provides the best values obtained to date). (+)-HRMS: *m/z* 918.9620. Calcd. for $C_{32}H_{39}GeN_6O_5Ru_2Si_2$: *m/z* 918.9909 [*M* – 2 CO + MeOH + MeCN + H]$^+$. IR (toluene): ν_{CO} = 2067 (m), 1998 (vs), 1982 (vs), 1965 (m), 1922 (m) cm^{-1}. ^{1}H NMR (CD_2Cl_2, 400.1 MHz, 298 K): δ 9.20 (dd, *J* = 4.8 and 1.4 Hz, 1 H), 8.81 (dd, *J* = 3.9 and 2.0 Hz, 1 H), 8.71 (s, 1 H, NC*H*N), 8.23 (t, *J* = 7.4 Hz, 2 H), 7.82 (dd, *J* = 16.0 and 8.0 Hz, 2 H), 7.64 (dd, *J* = 16.0 and 8.0 Hz, 2 H), 7.56–7.40 (m, 4 H), 0.35 (s, 9 H, Si(C*H*$_3$)$_3$), –0.10 (s, 9 H, Si(C*H*$_3$)$_3$) ppm. ^{13}C{^{1}H} NMR (CD_2Cl_2, 100.6 MHz, 298 K): δ 204.5 (s, *C*O), 203.7 (s, 4 *C*O), 201.9 (s, CO), 156.8 (s, *C*H), 155.3 (s, *C*H), 150.4 (s, *C*H), 147.5 (s, *C*), 146.2 (s, *C*), 144.5 (s, *C*), 141.2 (s, *C*), 137.2 (s, *C*H), 136.4 (s, *C*H), 130.0 (s, *C*), 129.8 (s, *C*),

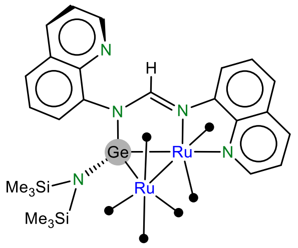

(**6a**)

128.1 (s, *C*H), 127.4 (s, *C*H), 126.7 (s, *C*H), 126.3 (s, *C*H), 123.2 (s, *C*H), 122.3 (s, *C*H), 121.9 (s, *C*H), 115.4 (s, *C*H), 5.9 (Si(CH_3)$_3$), 5.2 (Si(CH_3)$_3$) ppm.

[Ru_2{μ_{Sn}-κ^3*Sn,N,N'*-Sn(hmds)(bqfam)}$(CO)_6$] (6b): [$Ru_3(CO)_{12}$] (64 mg, 0.1 mmol) was added to a solution of stannylene **1b** (86 mg, 0.15 mmol) in toluene (5 mL) in a J. Young ampoule to give a dark red suspension, which was heated at 60 °C for 1.5 h (the ampoule was connected open to a Schlenk-vacuum line). The solvent of the resultant suspension was evaporated *in vacuo* and the resulting residue was washed with hexane (2 mL) and vacuum-dried to give **6b** as a dark wine solid (117 mg, 82%). Anal. (%) calcd. for $C_{31}H_{31}N_5O_6Ru_2Si_2Sn$ (*M* = 946.62 amu): C, 39.33; H, 3.30; N, 7.40; found: C, 38.40; H, 2.45; N, 7.34 (possibly affected by the air-sensitivity of the compound, this analysis provides the best values obtained to date). (+)-HRMS: *m/z* 976.9285. Calcd. for $C_{32}H_{51}N_6O_5Ru_2Si_2Sn$: *m/z* 977.06541 [M – 5 CO + MeCN + 4 MeOH + H]$^+$. IR (toluene, cm^{-1}): ν_{CO} = 2061 (m), 2014 (m), 1992 (vs), 1975 (s), 1963 (s), 1914 (m). ^{1}H NMR (CD_2Cl_2, 300.1 MHz, 298 K): δ 9.18 (d, *J* = 6.4 Hz, 1 H), 9.02 (d, *J* = 6.0 Hz, 1 H), 8.88 (s, 1 H, NC*H*N), 8.29 (d, *J* = 9.9 Hz, 1 H), 8.18 (d, *J* = 10.0 Hz, 1 H), 7.80–7.40 (m, 9 H), 0.06 (br s, 18 H, 2 Si(CH_3)$_3$) ppm. ^{13}C{^{1}H} NMR (CD_2Cl_2, 100.6 MHz, 298 K): δ 155.5 (s, N*C*HN), 155.3 (s, *C*H), 149.0 (s, *C*H), 148.1 (s, *C*), 146.0 (s, *C*), 142.2 (s, *C*), 141.8 (s, *C*), 137.4 (s, *C*H), 136.7 (s, *C*H), 130.0 (s, *C*), 129.5 (s, *C*), 128.0 (s, *C*H), 127.6 (s, *C*H), 124.3 (s, *C*H), 123.3 (s, *C*H), 122.4 (s, 2 *C*H), 121.5 (s, *C*H), 115.1 (s, *C*H), 5.8 (br s, Si(CH_3)$_3$), 3.8 (br s, Si(CH_3)$_3$) ppm. ^{119}Sn{^{1}H} NMR (CD_2Cl_2, 149.2 MHz, 298 K): not found.

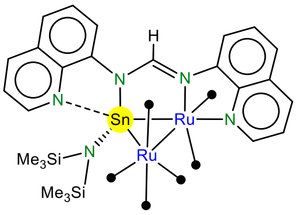

(**6b**)

[Co_2{μ_{Ge}-κ^3*Ge,N,N'*-Ge(hmds)(bqfam)}(μ-CO)$(CO)_4$] (7a): [$Co_2(CO)_8$] (34 mg, 0.1 mmol,) was added to a solution of germylene **1a** (53 mg, 0.1 mmol) in toluene (5 mL) in a J. Young ampoule to give a dark brown suspension, which was heated at 60 °C for 2 h (the ampoule was connected open to a Schlenk-vacuum line). The solvent of the resulting suspension was evaporated *in vacuo* and the resulting residue was washed with hexane (4 × 2 mL) and vacuum-dried to give **7a** as a brown solid (71 mg, 90%). Anal. (%) calcd. for $C_{30}H_{31}Co_2GeN_5O_5Si_2$ (*M* = 788.26 amu): C, 45.71; H, 3.96; N, 8.89; found: C, 45.79; H, 3.68; N, 8.79. (+)-HRMS: The molecular ion (M$^+$) and/or its fragments could not be identified in the obtained spectra. IR (toluene): ν_{CO} = 2032 (s), 1972 (vs), 1965 (vs), 1801

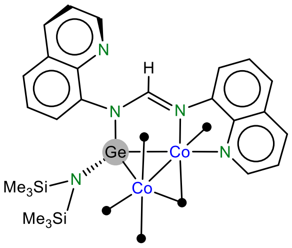

(**7a**)

(m, br) cm^{-1}. ^{1}H (CD_2Cl_2, 400.1 MHz, 298 K): δ 9.58 (s, 1 H, NC*H*N), 9.19 (d, *J* = 4.7 Hz, 1 H), 9.02 (d, *J* = 3.8 Hz, 1 H), 8.31 (d, *J* = 8.2 Hz, 1 H), 8.20 (d, *J* = 8.4 Hz, 1 H), 7.89–7.50 (m, 8 H), 0.36 (s, 9 H, Si(C*H*$_3$)$_3$), –0.26 (s, 9 H, Si(C*H*$_3$)$_3$) ppm. $^{13}C\{^{1}H\}$ NMR (CD_2Cl_2, 100.6 MHz, 298 K): δ 205.9 (*C*Os), 156.2 (s, N*C*HN), 153.1 (s, *C*H), 150.1 (s, *C*H), 146.5 (s, *C*), 145.9 (s, *C*), 142.4 (s, *C*), 139.1 (s, *C*), 137.0 (s, *C*H), 136.5 (s, *C*H), 130.2 (s, *C*), 129.8 (s, *C*), 128.4 (s, *C*H), 126.8 (s, *C*H), 125.3 (s, *C*H), 125.0 (s, *C*H), 123.5 (s, *C*H), 122.7 (s, *C*H), 122.0 (s, *C*H), 115.0 (s, *C*H), 5.0 (Si(*C*H$_3$)$_3$), 3.9 (Si(*C*H$_3$)$_3$) ppm.

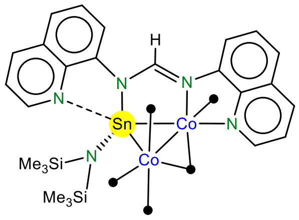

(**7b**)

[Co$_2$\{μ$_{Sn}$-κ^3*Sn,N,N'*-Sn(HMDS)(bqfam)\}(μ-CO)(CO)$_4$] (7b): [$Co_2(CO)_8$] (34 mg, 0.1 mmol, 0.2 mmol Co) was added to a solution of stannylene **1b** (58 mg, 0.1 mmol) in toluene (5 mL) in a J. Young ampoule to give a dark brown suspension, which was heated at 60 °C for 2 h (the ampoule was connected open to a Schlenk-vacuum line). The solvent of the resultant suspension was evaporated *in vacuo* and the resulting residue was washed with hexane (2 mL) and vacuum-dried to give **7b** as a brown solid (68 mg, 82%). Anal. (%) calcd. for $C_{30}H_{31}Co_2N_5O_5Si_2Sn$ (*M* = 834.35 amu): C, 43.19; H, 3.74; N, 8.39; found: C, 41.60; H, 3.23; N, 7.66 (possibly affected by the air-sensitivity of the compound, this analysis provides the best values obtained to date). (+)-HRMS: The molecular ion (M^+) and/or its fragments could not be identified in the obtained spectra. IR (toluene, cm^{-1}): ν_{CO} = 2015 (m), 1955 (m), 1945 (vs, br). ^{1}H (CD_2Cl_2, 400.5 MHz, 298 K): δ 9.51 (s, 1 H, NC*H*N), 9.13 (br s, 1 H), 8.96 (br s, 1 H), 8.52 (d, *J* = 6.2 Hz, 1 H), 8.12 (d, *J* = 7.4 Hz, 1 H), 7.95–7.35 (m, 8 H), 0.37 (s, 9 H, Si(C*H*$_3$)$_3$), –0.35 (s, 9 H, Si(C*H*$_3$)$_3$). $^{13}C\{^{1}H\}$ NMR (CD_2Cl_2, 100.7 MHz, 298 K): δ 153.3 (s, N*C*HN), 152.4 (s, *C*H), 147.1 (s, *C*H), 146.9 (s, *C*), 145.8 (s, *C*), 140.8 (s, *C*), 140.2 (s, *C*H), 138.3 (s, *C*), 135.4 (s, *C*H), 132.7 (s, *C*), 129.8 (C), 129.5 (s, *C*H), 128.1 (s, *C*H), 123.6 (s, *C*H), 123.4 (s, *C*H), 123.0 (s, *C*H), 121.6 (s, *C*H), 115.5 (s, *C*H), 114.7 (s, *C*H), 6.0 (Si(*C*H$_3$)$_3$), 4.3 (Si(*C*H$_3$)$_3$) ppm. $^{119}Sn\{^{1}H\}$ NMR (CD_2Cl_2, 149.2 MHz, 298 K): not found.

X-ray Diffraction Analyses. Crystals of **1b**, **3a**, **4a**,·**4b**, **5a**·and **6a** were analyzed by X-ray diffraction. A selection of crystal, measurement and refinement data is given in Table S1. Diffraction data were collected on a Bruker D8 Venture Photon III-14 (with MoKα radiation for **1b**) and Oxford Diffraction Xcalibur Onyx Nova Geminy (with CuKα radiation for **4a**, **4a**,·**4b**, **5a**·and **6a**) single crystal diffractometers. Empirical absorption

corrections were applied using SADABS-2016/2[S6] (for **1b**) and the SCALE3 ABSPACK algorithm as implemented in Chrysalis RED[S7] (for **3a**, **4a**,·**4b**, **5a**·and **6a**). The structures were solved with SIR-97.[S8] Isotropic and full matrix anisotropic least square refinements were carried out using SHELXL.[S9] One $SiMe_3$ group (Si1) of the hmds moiety of **1b** was disordered over two positions with a 73:27 occupancy ratio. Several high resolution reflections were left out from the refinement of **4a** (–3 10 10, –4 9 10, –3 9 12 and –3 9 11), **5a** (–3 10 10 and –3 9 12) and **6a** (1 7 19, 0 5 19, 4 4 18 and –5 10 16) because their intensities, likely affected by some unresolved twinning, showed high S values. The low resolution reflection –4 –1 4 was left out from the refinement of **5a** because its intensity, likely affected by the beamstop, showed high S value. The WINGX program system[S10] was used throughout the structure determinations. The molecular plots were made with MERCURY.[S11]

Theoretical Calculations. Structure optimizations were performed with the *Gaussian09* suite of programs,[S12] using the wB97xd functional,[S13] which includes the second generation of Grimme's dispersion interaction correction.[S14] The Stuttgart-Dresden relativistic effective core potentials and the associated basis sets (SDD) were used for the Ge, Sn and Ru atoms.[S15] The basis set used for the remaining atoms was the cc-pVDZ.[S16] Frequency calculations confirmed the optimized structures as energy minima (zero imaginary eigenvalues) or as transition states (one imaginary eigenvalue). Gibbs energies were computed at 298.15 K and 1.0 atm. Solvation free energies were obtained with the selfconsistent reaction field (SCRF) for the standard continuum solvation model (CPCM),[S17] by using the single-point solvation energy of the optimized structures and the thermodynamic correction from the gas phase calculations.

References

S1 Cabeza, J. A.; Reynes, J. F.; García, F.; García-Álvarez, P.; García-Soriano, R. Fast and scalable solvent-free access to Lappert's heavier tetrylenes E{N(SiMe3)2}2 (E = Ge, Sn, Pb) and ECl{N(SiMe3)2} (E = Ge, Sn). *Chem. Sci.* **2023**, *14*, 12477–12483.

S2 Yamaguchi, Y.; Yamanishi, K.; Kondo, M.; Tsukada, N. Synthesis of Dinuclear (μ-η3-Allyl)palladium(I) and -platinum(I) Complexes Supported by Chelate-Bridging Ligands. Organometallics 2013, 32, 4837–4842.

S3 Mathews, C. J.; Smith, P. J.; Welton, T. Novel palladium imidazole catalysts for Suzuki cross-coupling reactions. *J. Mol. Catal. A. Chem*. **2003**, *206*, 77–82.

S4 Drew, D.; Doyle, J. R. Cyclic Diolefin Complexes of Platinum and Palladium. *Inorg. Synth.* **1972**, *13*, 47–55.

S5 Usón, R.; Laguna, A.; Laguna, M.; Briggs, D. A.; Murray, H. H.; Fackler, J. (Tetrahydrothiophene)Gold(I) or Gold(III) Complexes. *Inorg. Synth*. **1989**, *26*, 85–91.

S6 *SADABS-2016/2*: Krause, L.; Herbst-Irmer, R.; Sheldrick, G. M.; Stalke, D. Comparison of silver and molybdenum microfocus X-ray sources for single-crystal structure determination. *J. Appl. Crystallogr.* **2015**, *48,* 3–10.

S7 *CrysAlisPro RED*, version 1.171.38.46: Oxford Diffraction Ltd., Oxford, UK, 2015.

S8 *SIR-97*: Altomare, A.; Burla, M. C.; Camalli, M.; Cascarano, G. L.; Giacovazzo, C.; Guagliardi, A.; Moliterni, A. G. C.; Polidori, G.; Spagna, R. SIR97: a new tool for crystal structure determination and refinement. *J. Appl. Crystallogr.* **1999**, *32*, 115–119.

S9 *SHELXL-2014*: Sheldrick, G. M. A short history of SHELX. *Acta Cryst.* **2008**, *A64*, 112–122.

S10 *WINGX*, version 2021.3: Farrugia, L. WinGX and ORTEP for Windows: an update. *J. Appl. Crystallogr.* **2012**, *45,* 849–854.

S11 *MERCURY,* version 2022.2.0 (build 353591): Cambridge Crystallographic Data Centre, Cambridge, UK, 2022.

S12 Frisch, M. J.; Trucks, G. W.; Schlegel, H. B.; Scuseria, G. E.; Robb, M. A.; Cheeseman, J. R.; Scalmani, G.; Barone, V.; Mennucci, B.; Petersson, G. A.;

Nakatsuji, H.; Caricato, M.; Li, X.; Hratchian, H. P.; Izmaylov, A. F.; Bloino, J.; Zheng, G.; Sonnenberg, J. L.; Hada, M.; Ehara, M.; Toyota, K.; Fukuda, R.; Hasegawa, J.; Ishida, M.; Nakajima, T.; Honda, Y.; Kitao, O.; Nakai, H.; Vreven, T.; Montgomery, J. A., Jr.; Peralta, J. E.; Ogliaro, F.; Bearpark, M.; Heyd, J. J.; Brothers, E.; Kudin, K. N.; Staroverov, V. N.; Kobayashi, R.; Normand, J.; Raghavachari, K.; Rendell, A.; Burant, J. C.; Iyengar, S. S.; Tomasi, J.; Cossi, M.; Rega, N.; Millam, J. M.; Klene, M.; Knox, J. E.; Cross, J. B.; Bakken, V.; Adamo, C.; Jaramillo, J.; Gomperts, R.; Stratmann, R. E.; Yazyev, O.; Austin, A. J.; Cammi, R.; Pomelli, C.; Ochterski, J. W.; Martin, R. L.; Morokuma, K.; Zakrzewski, V. G.; Voth, G. A.; Salvador, P.; Dannenberg, J. J.; Dapprich, S.; Daniels, A. D.; Farkas, O.; Foresman, J. B.; Ortiz, J. V.; Cioslowski, J.; Fox, D. J. *Gaussian 09*, revision A.01; Gaussian, Inc.: Wallingford, CT, 2009.

S13 Chai, J.-D.; Head-Gordon, M. Long-range corrected hybrid density functionals with damped atom–atom dispersion corrections. *Phys. Chem. Chem. Phys*. **2008**, *10*, 6615–6620.

S14 (a) Ehrlich, S.; Moellmann, J.; Grimme, S. Dispersion-Corrected Density Functional Theory for Aromatic Interactions in Complex Systems. *Acc. Chem. Res*. **2013**, *46*, 916–926. (b) Grimme, S. Density functional theory with London dispersion corrections. *Comp. Mol. Sci.* **2011**, *1*, 211–228; (c) Schwabe, T.; Grimme, S. Theoretical Thermodynamics for Large Molecules: Walking the Thin Line between Accuracy and Computational Cost. *Acc. Chem. Res*. **2008**, *41*, 569–579.

S15 (a) Dolg, M.; Wedig U.; Stoll, H.; Preuss, H. Energy-adjusted ab initio pseudopotentials for the first row transition elements. *J. Chem. Phys.* **1987**, 86, 866–872. (b) Martin, J. M. L.; Sundermann, A. Correlation consistent valence basis sets for use with the Stuttgart–Dresden–Bonn relativistic effective core potentials: The atoms Ga–Kr and In–Xe. *J. Chem. Phys.* **2001**, 114, 3408–3420.

S16 Dunning, T. H. Gaussian basis sets for use in correlated molecular calculations. I. The atoms boron through neon and hydrogen. *J. Chem. Phys*. **1989**, *90*, 1007–1023.

S17 (a) Barone, V.; Cossi, M. Quantum Calculation of Molecular Energies and Energy Gradients in Solution by a Conductor Solvent Model. *J. Phys. Chem. A* **1998**, *102*, 1995−2001. (b) Cossi, M.; Rega, N.; Scalmani, G.; Barone, V. Energies, structures,

and electronic properties of molecules in solution with the C-PCM solvation model. *J. Comput. Chem.* **2003**, *24*, 669−681.

Table S1. Crystal, measurement and refinement data for the compounds studied by X-ray diffraction.

	**1b**	**3a**	**4a**	**4b**	**5a**	**6a**
formula	$C_{25}H_{31}N_5Si_2Sn$	$C_{25}H_{31}AuClGeN_5Si_2$	$C_{25}H_{31}Cl_2GeN_5PdSi_2$	$C_{25}H_{31}Cl_2N_5PdSi_2Sn$	$C_{25}H_{31}Cl_2GeN_5PtSi_2$	$C_{25}H_{31}GeN_5O_6Ru_2Si_2$
fw	576.42	762.73	707.62	753.72	796.31	900.52
cryst syst	triclinic	orthorhombic	triclinic	monoclinic	triclinic	monoclinic
space group	*P*–1	*P*$_{212121}$	*P*–1	*P*21/c	*P*–1	*P*21/n
a, Å	8.1012(7)	12.7054(8)	9.9576(8)	14.4066(2)	9.9263(5)	9.0852(2)
b, Å	12.341(1)	13.8028(1)	11.2596(7)	12.1228(1)	11.2466(5)	24.2964(4)
c, Å	14.123(1)	16.4011(1)	14.638(1)	17.1752(3)	14.6844(6)	16.1867(3)
α, deg	82.125(4)	90	72.183(6)	90	72.402(4)	90
β, deg	73.355(3)	90	78.029(6)	107.175(2)	78.307(4)	90.475(2)
γ, deg	82.926(3)	90	71.960(6)	90	72.748(4)	90
V, Å^3	4434.6(2)	2876.3(2)	1474.1(2)	2865.86(7)	1480.8(1)	3572.9(1)
Z	2	4	2	4	2	4
F(000)	588	1488	712	1496	776	1792
D_{calcd}, g cm^{-3}	1.434	1.761	1.594	1.747	1.786	1.674
μ, mm^{-1}	1.069 (MoKα)	12.572 (CuKα)	8.811 (CuKα)	14.705 (CuKα)	12.580 (CuKα)	8.803 (CuKα)
cryst size, mm	0.15 x 0.14 x 0.04	0.26 x 0.16 x 0.12	0.33 x 0.26 x 0.07	0.29 x 0.20 x 0.17	0.21 x 0.19 x 0.14	0.25 x 0.15 x 0.15
T, K	123(2)	150(2)	150(2)	150(2)	150(2)	150(2)
θ range, deg	2.37 to 30.51	4.19 to 69.58	4.28 to 69.35	3.21 to 69.57	3.18 to 69.54	3.28 to 69.72
min./max. *h*, *k*, *l*	–11/11, –17/17, –20/20	–10/15, –15/16, –18/19	–11/11, –10/13, –17/16	–16/17, –10/14, –20/20	–11/11, –13/11, –17/17	–10/10, –29/27, –19/19
no. collected reflns	39454	15201	12716	14071	13005	20707
no. unique reflns	8142	5230	5442	5324	5472	6640
no. reflns with $I > 2\sigma(I)$	7406	5142	5037	4805	5132	6242
no. params/restraints	344/0	322/0	331/0	331/0	331/0	430/0
GOF (on F^2)	1.049	1.068	1.087	1.060	1.051	1.041
R_1 (on F, $I > 2\sigma(I)$)	0.025	0.021	0.048	0.039	0.036	0.028
wR_2 (on F^2, all data)	0.064	0.053	0.139	0.111	0.098	0.075
min./max. $\Delta\rho$, e Å^{-3}	–0.573/0.683	–0.744/0.558	–1.041/1.017	–1.535/1.341	–1.550/1.189	–0.687/0.656
CCDC dep. no.	2309169	2309170	2309171	2309172	2309173	2309174

NMR Spectra

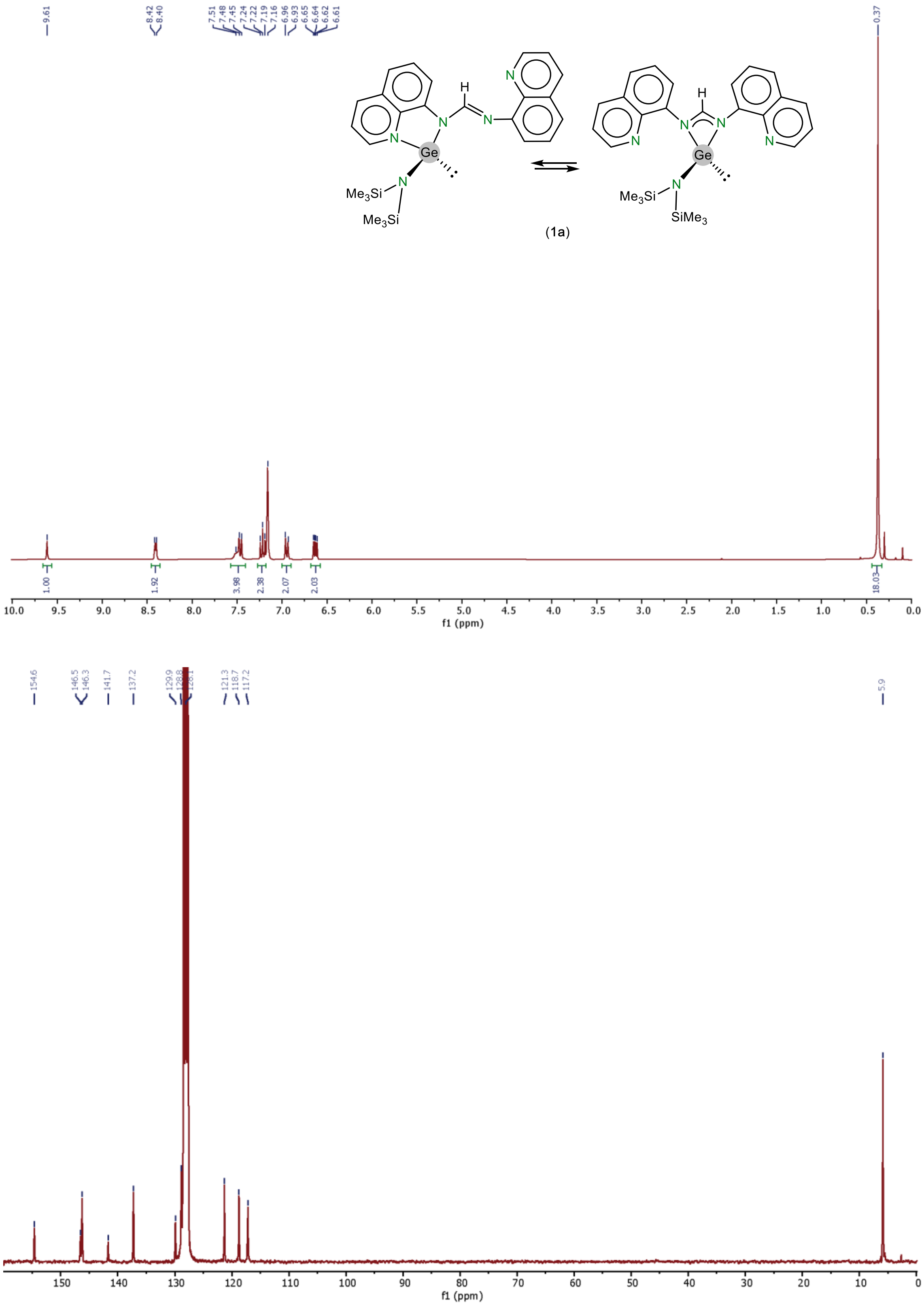

Figure S1. ^{1}H (top, 300.1 MHz) and $^{13}C\{^{1}H\}$ (bottom, 75.5 MHz) NMR spectra (C_6D_6, 298 K) of Ge(hmds)(bqfam) (**1a**).

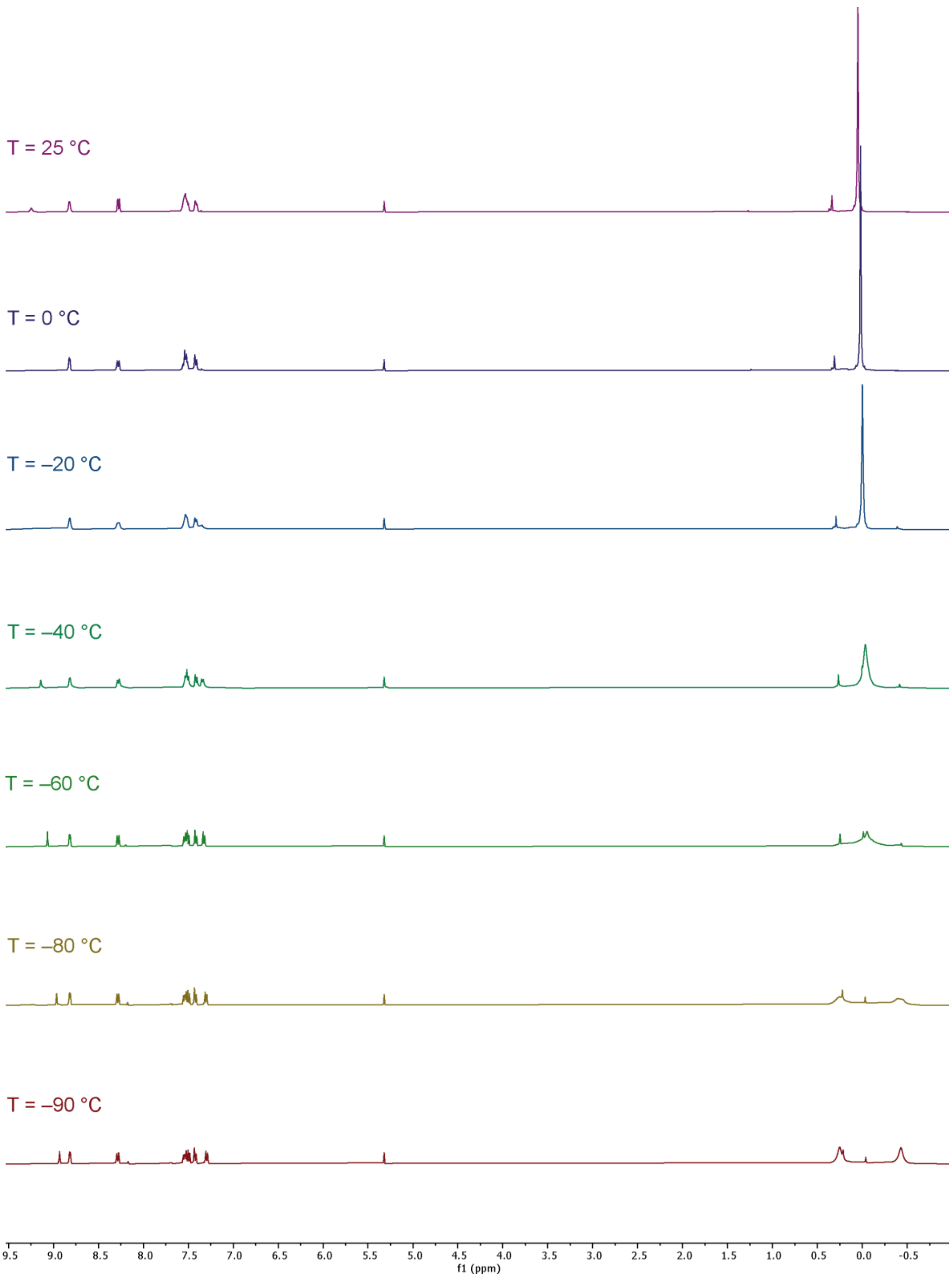

Figure S2. VT ^{1}H NMR spectra (CD_2Cl_2, 400.1 MHz) of **1a**.

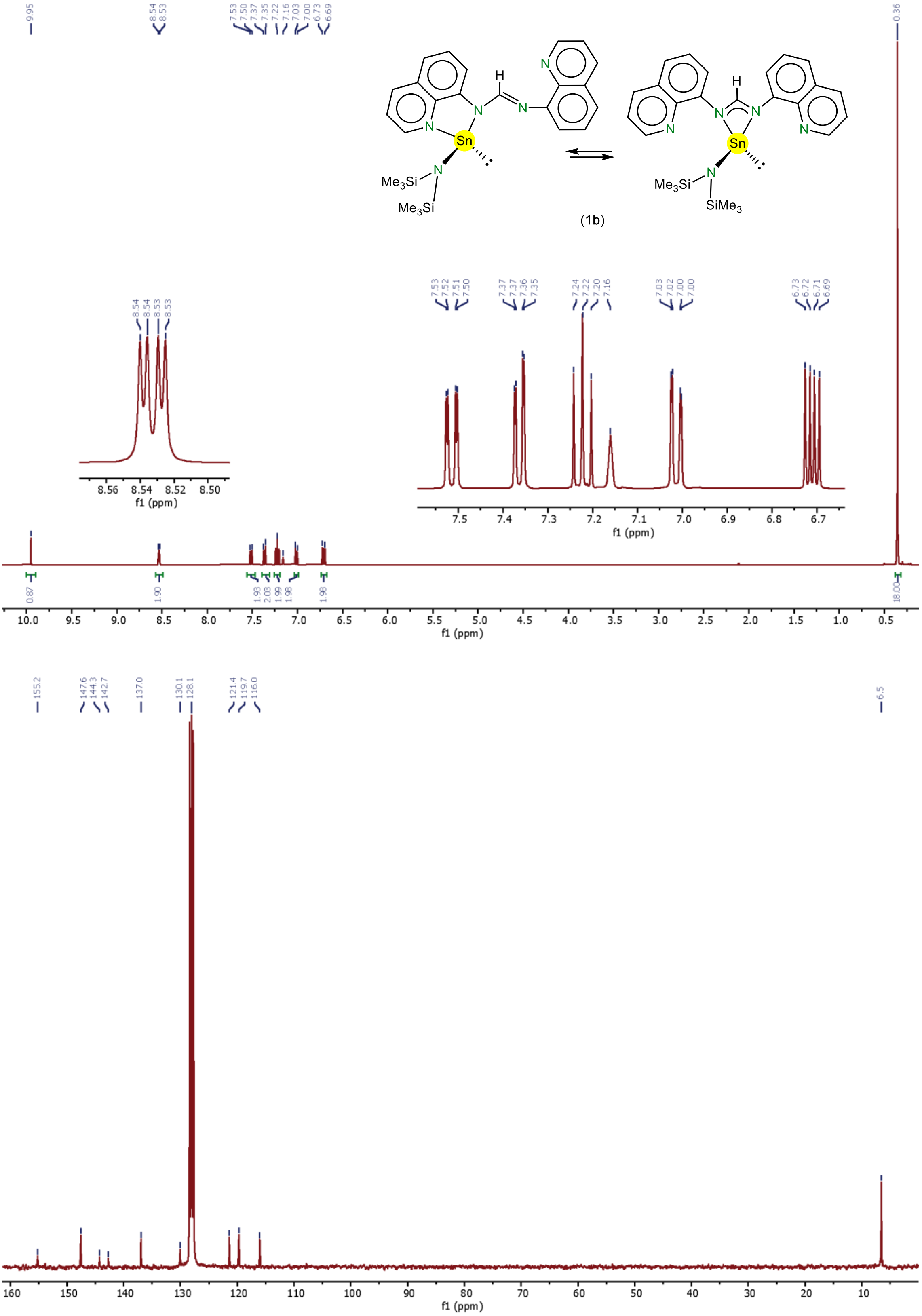

Figure S3. ^{1}H (top, 400.1 MHz) and $^{13}C\{^{1}H\}$ (bottom, 75.5 MHz) NMR spectra (C_6D_6, 298 K) of Sn(hmds)(bqfam) (**1b**).

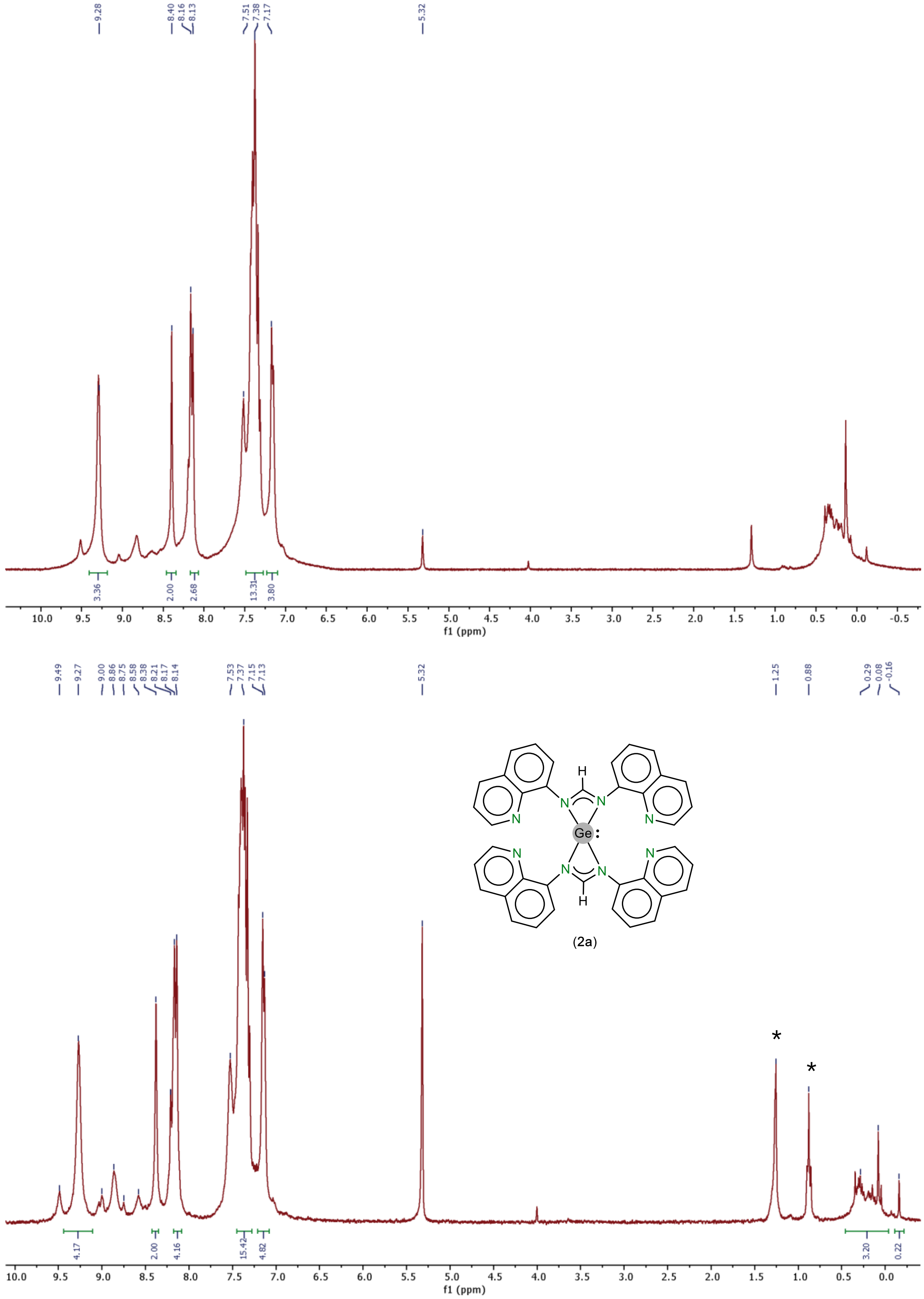

Figure S4. ^{1}H (top, 300.1 MHz) NMR spectra (CD_2Cl_2, 298 K) of the crude outcome of the reaction of Hbqfam with $Ge(hmds)_2$ in a 2:1 ratio (top) and of impurified isolated $Ge(bqfam)_2$ (**2a**). (* = *n*-hexane).

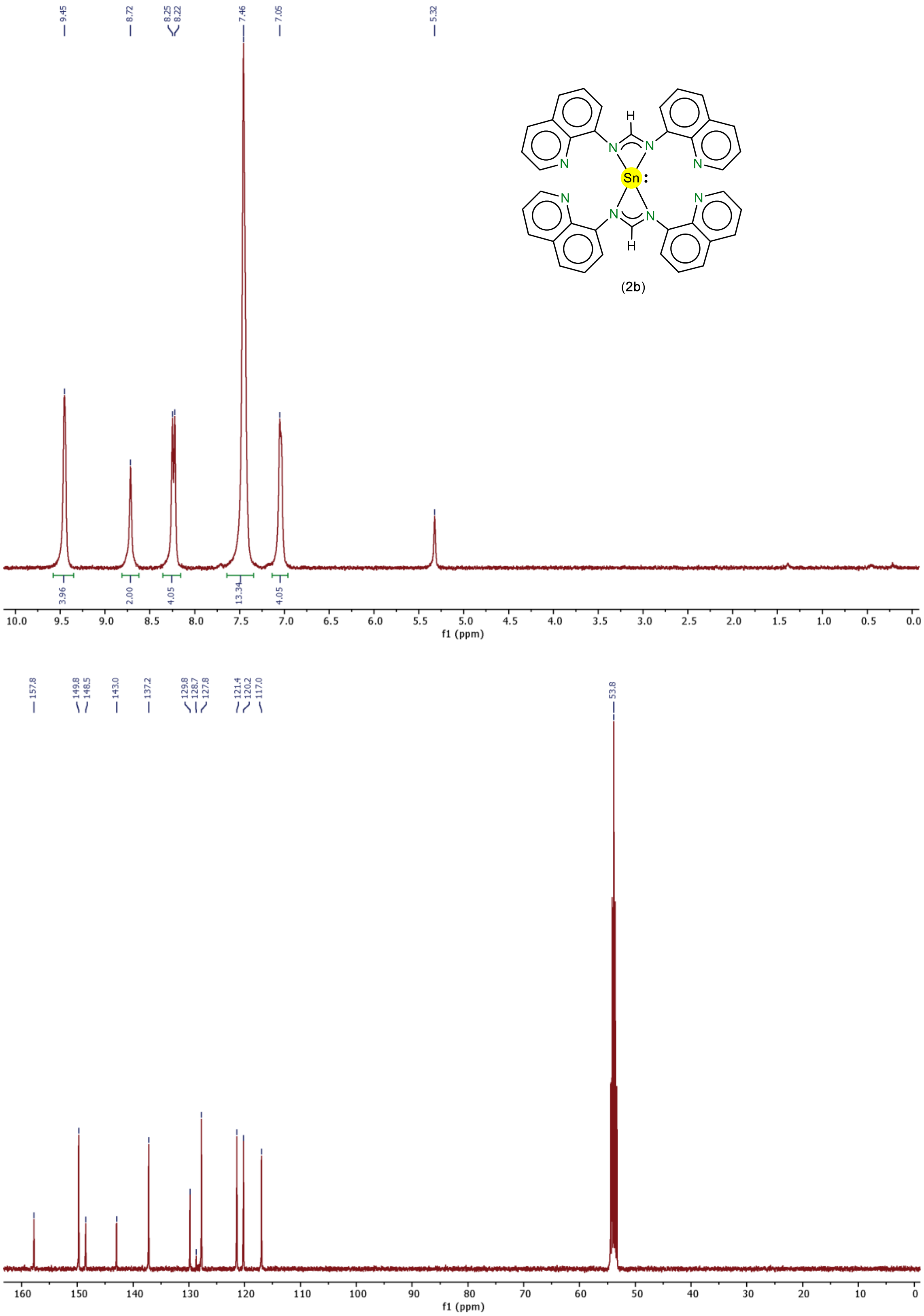

Figure S5. ^{1}H (top, 400.1 MHz) and $^{13}C\{^{1}H\}$ (bottom, 100.6 MHz) NMR spectra (CD_2Cl_2, 298 K) of $Sn(bqfam)_2$ (**2b**).

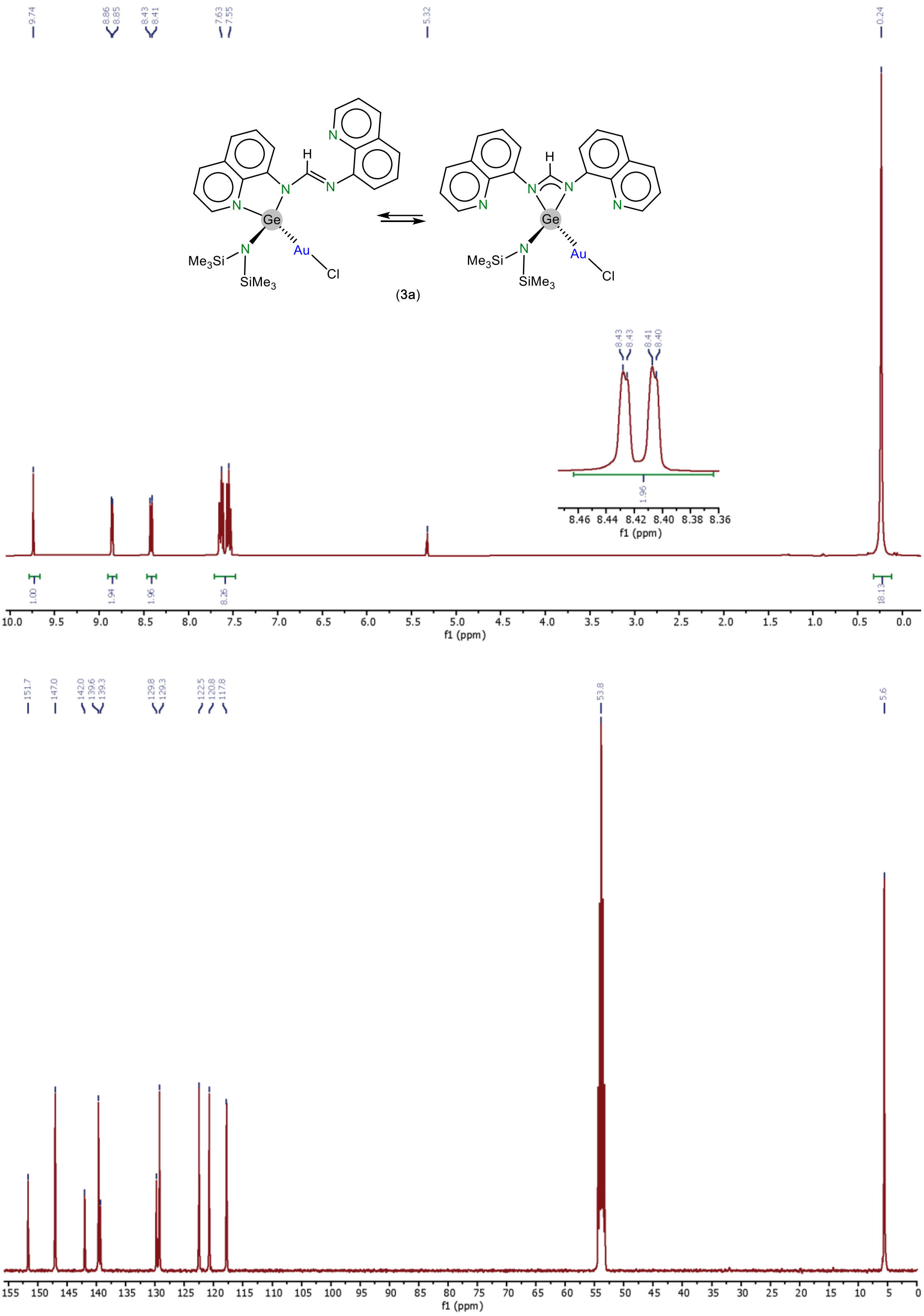

Figure S6. ^{1}H (top, 400.1 MHz) and $^{13}C\{^{1}H\}$ (bottom, 100.6 MHz) NMR spectra (CD_2Cl_2, 298 K) of [AuCl{κ^{1}*Ge*-Ge(hmds)(bqfam)}] (**3a**).

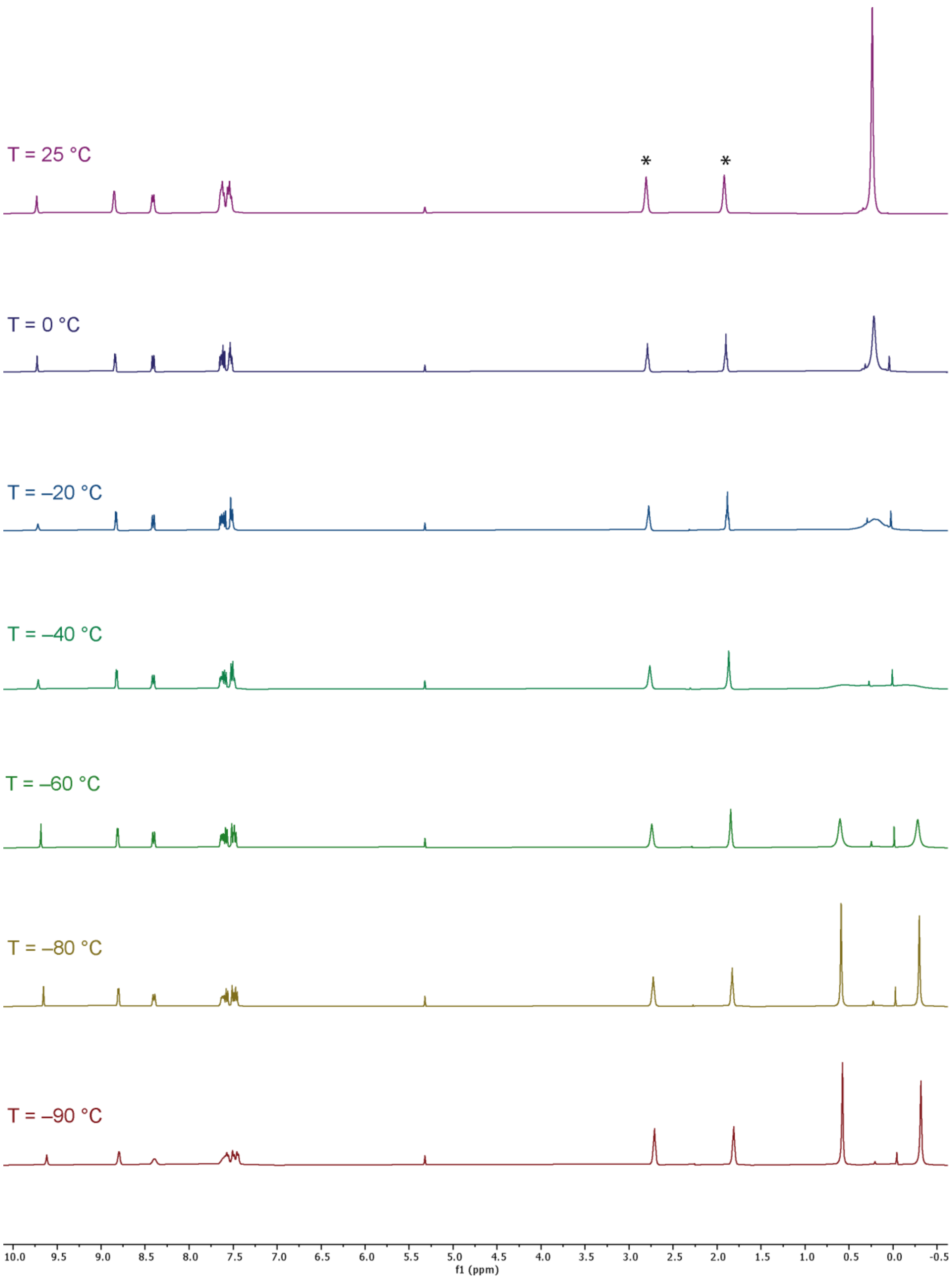

Figure S7. VT ^1H NMR spectra (CD_2Cl_2, 400.1 MHz) of the crude outcome of the reaction of germylene **1a** with [AuCl(tht)], showing the resonances of complex **3a** and tht (*).

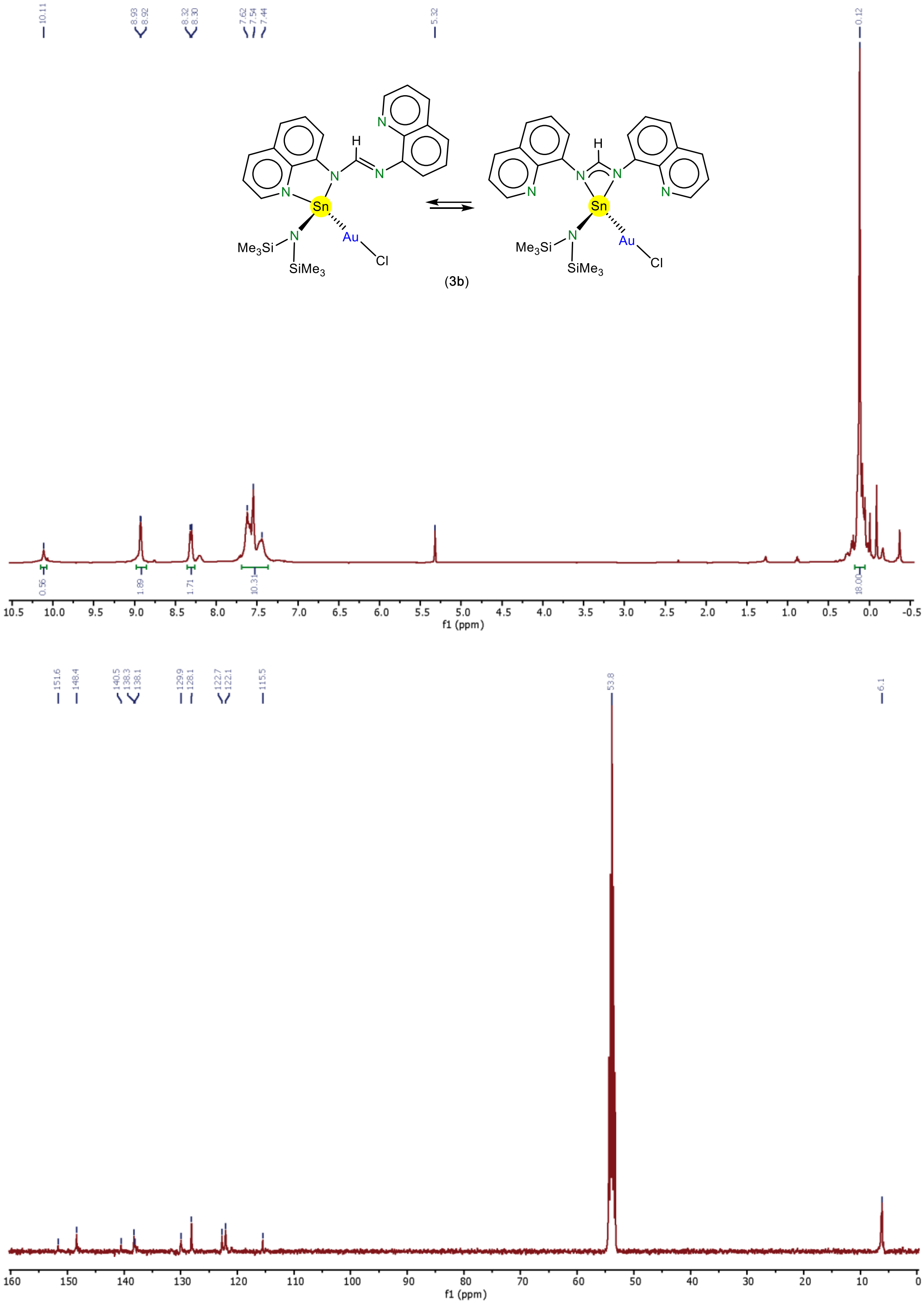

Figure S8. ^{1}H (top, 400.1 MHz) and $^{13}C\{^{1}H\}$ (bottom, 100.6 MHz) NMR spectra (CD_2Cl_2, 298 K) of [AuCl{κ^{1}*Sn*-Sn(hmds)(bqfam)}] (**3b**).

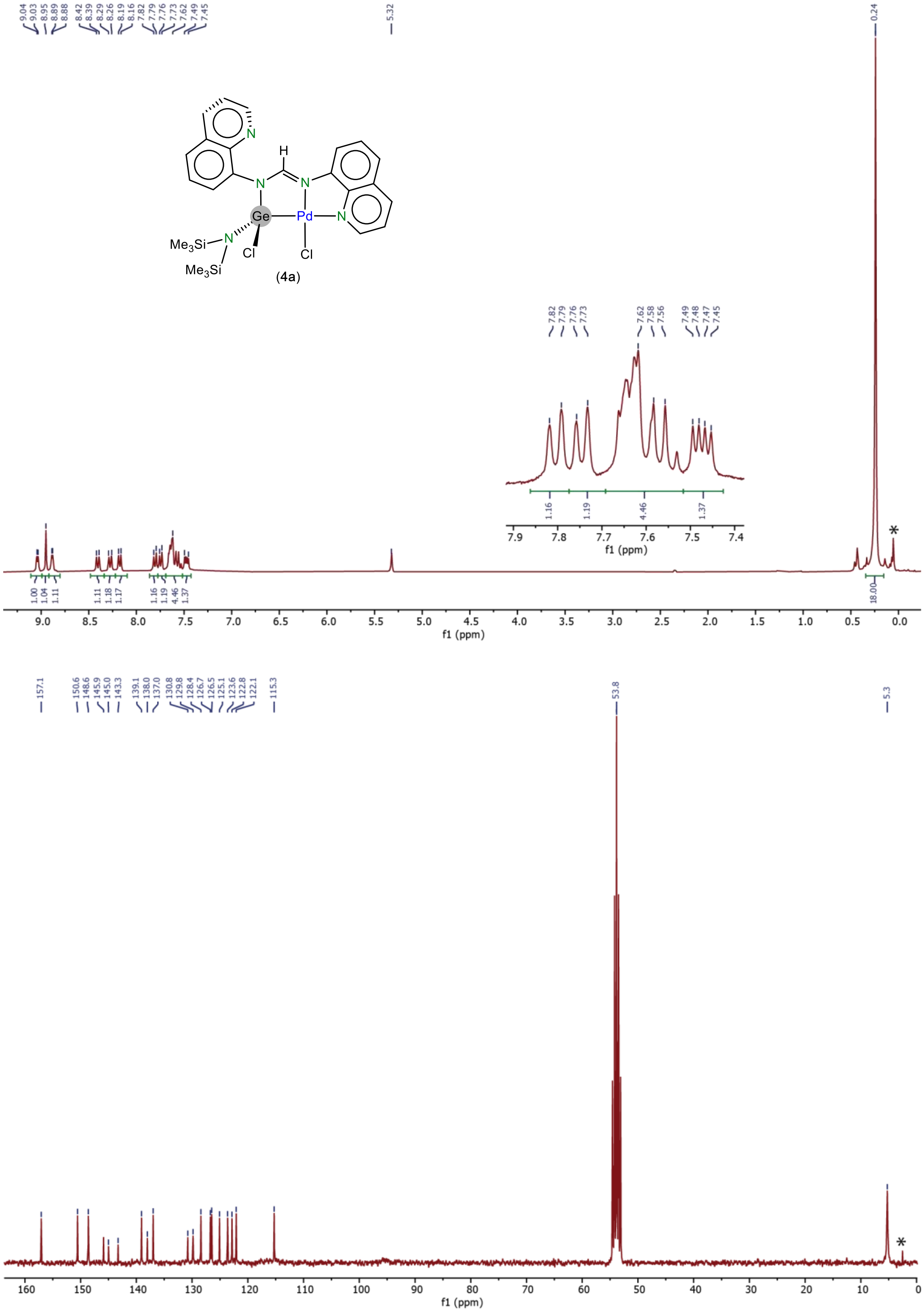

Figure S9. ^{1}H (top, 300.1 MHz) and $^{13}C\{^{1}H\}$ (bottom, 75.5 MHz) NMR spectra (CD_2Cl_2, 298 K) of [PdCl{κ^3*Ge*,*N*,*N'*-GeCl(hmds)(bqfam)}] (**4a**) (* = Hhmds).

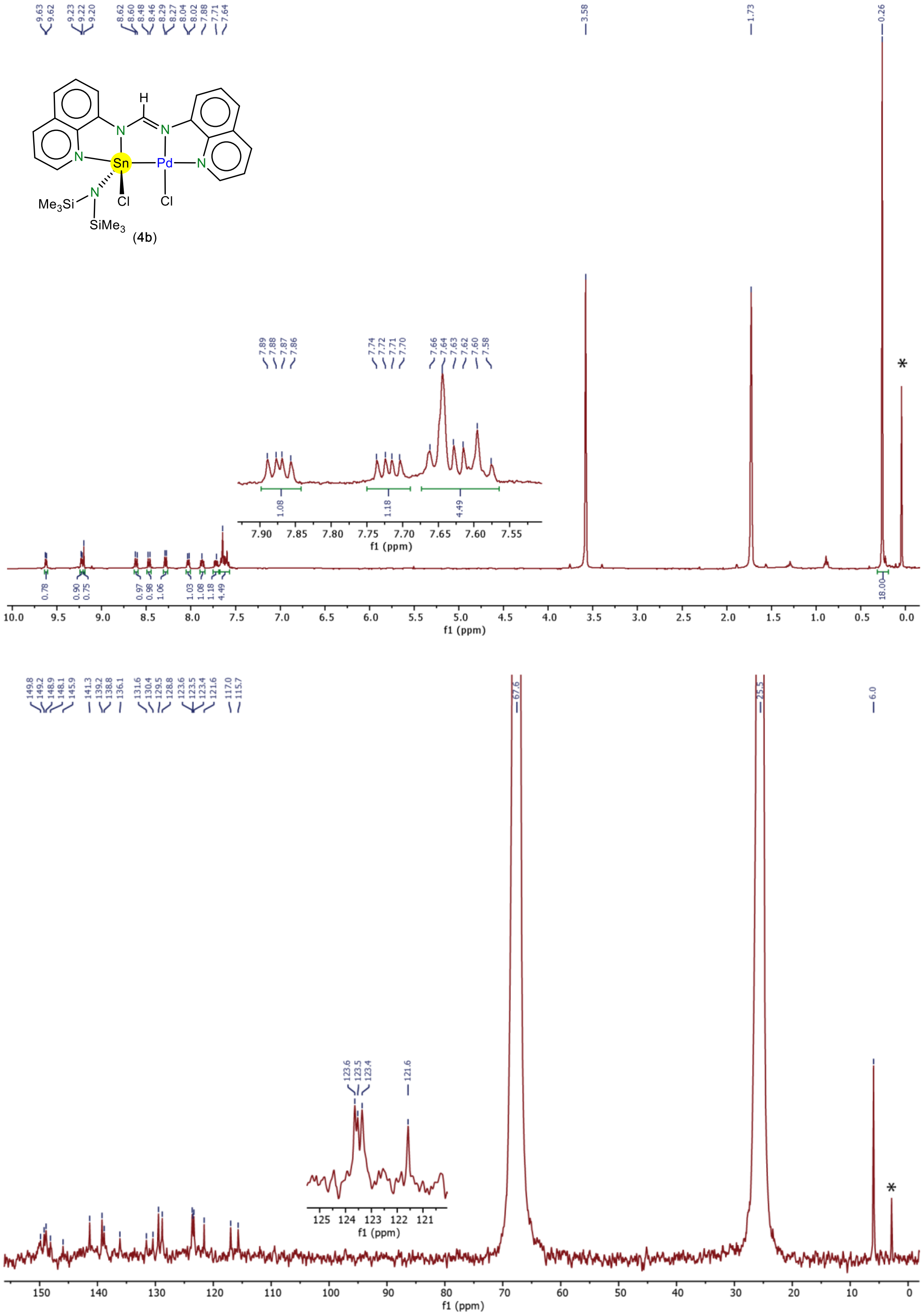

Figure S10. ^{1}H (top, 400.5 MHz) and ^{13}C{^{1}H} (bottom, 100.6 MHz) NMR spectra (THF-d_8, 298 K) of [PdCl{κ^3*Sn*,*N*,*N'*-SnCl(hmds)(bqfam)}] (**4b**) (* = Hhmds).

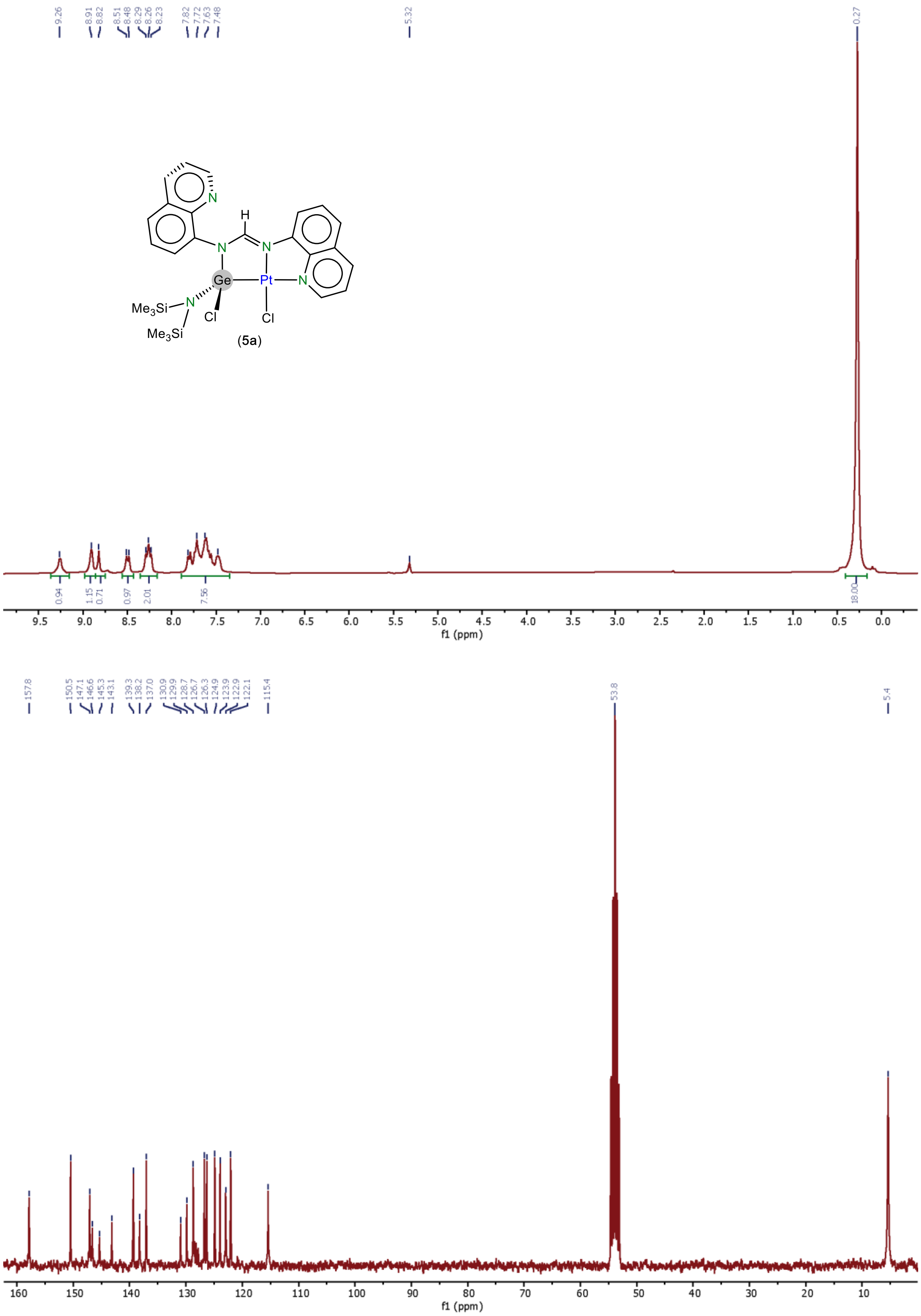

Figure S11. ^{1}H (top, 300.1 MHz) and $^{13}C\{^{1}H\}$ (bottom, 75.5 MHz) NMR spectra (CD_2Cl_2, 298 K) of [PtCl{κ^3*Ge*,*N*,*N'*-GeCl(hmds)(bqfam)}] (**5a**).

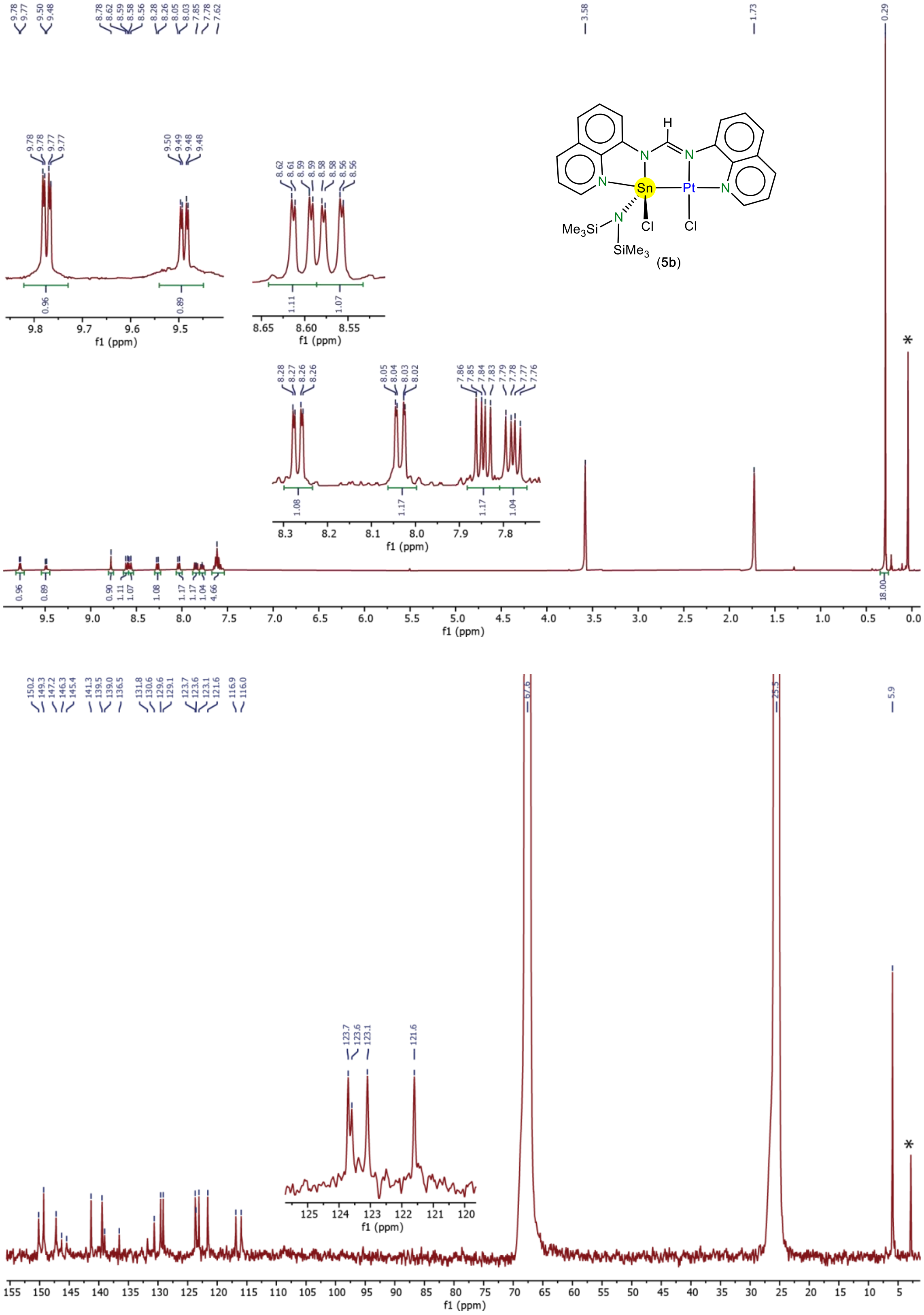

Figure S12. ^{1}H (top, 400.5 MHz) and ^{13}C{^{1}H} (bottom, 100.6 MHz) NMR spectra (THF-d_8, 298 K) of [PtCl{κ^{3}*Sn*,*N*,*N'*-SnCl(hmds)(bqfam)}] (**5b**) (* = Hhmds).

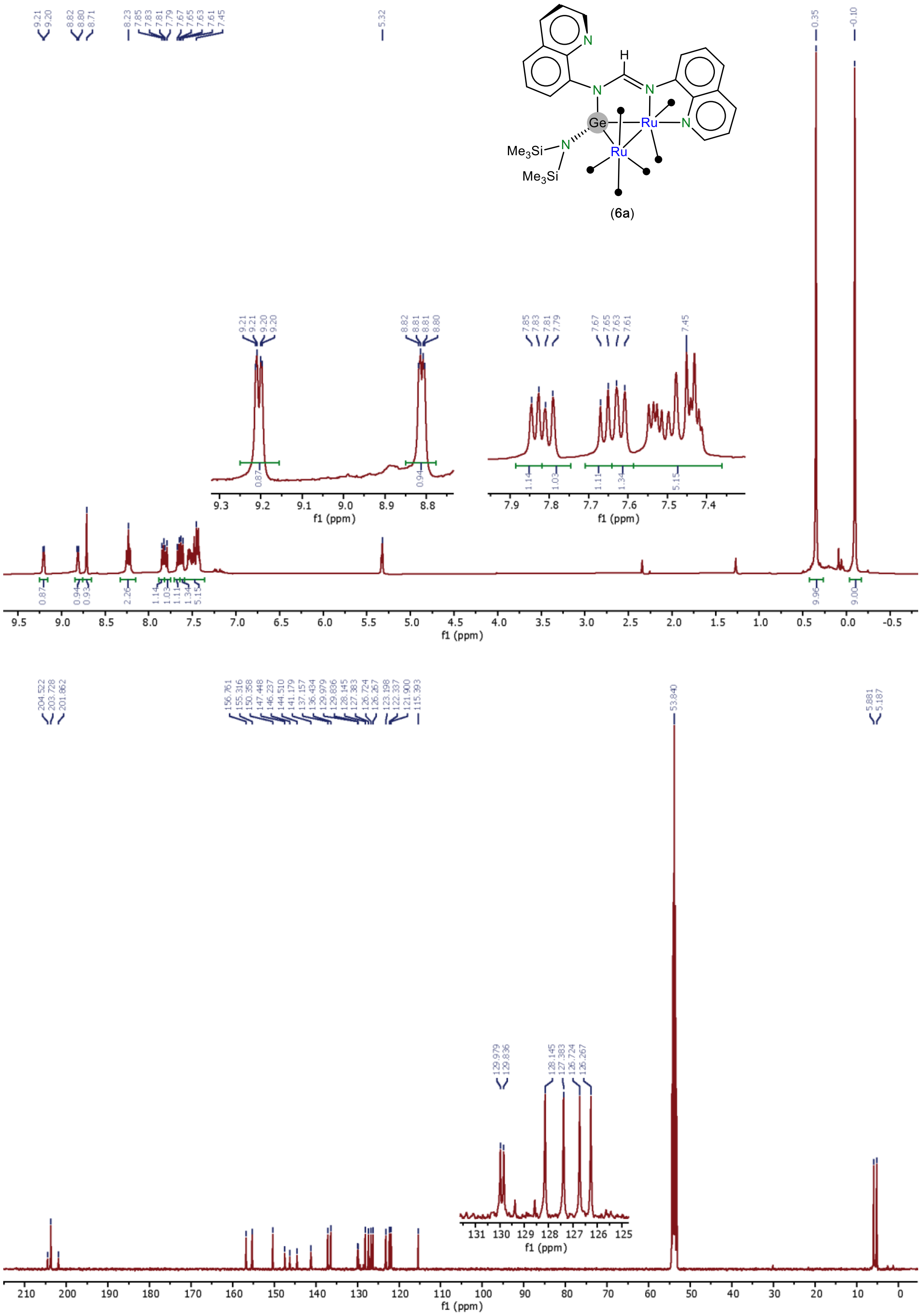

Figure S13. ^{1}H (top, 400.1 MHz) and $^{13}C\{^{1}H\}$ (bottom, 100.6 MHz) NMR spectra (CD_2Cl_2, 298 K) of [$Ru_2\{\mu_{Ge}$-κ^3*Ge,N,N'*-Ge(hmds)(bqfam)$\}(CO)_6$] (**6a**).

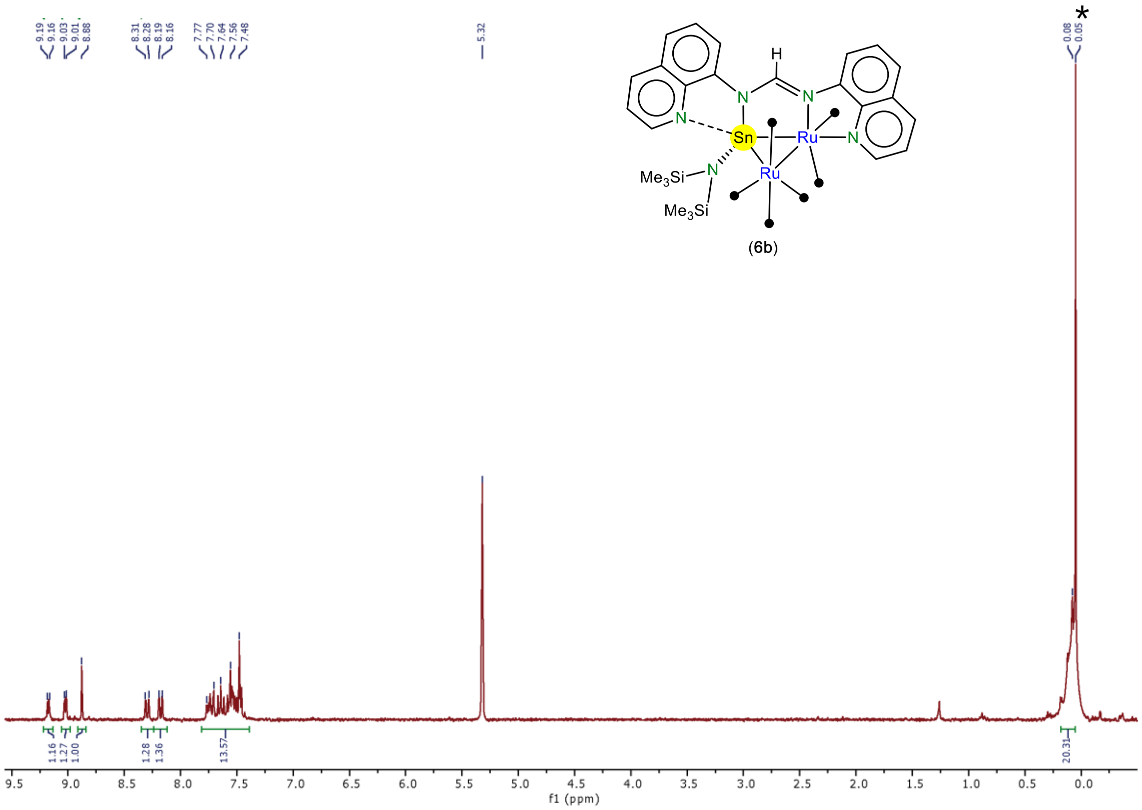

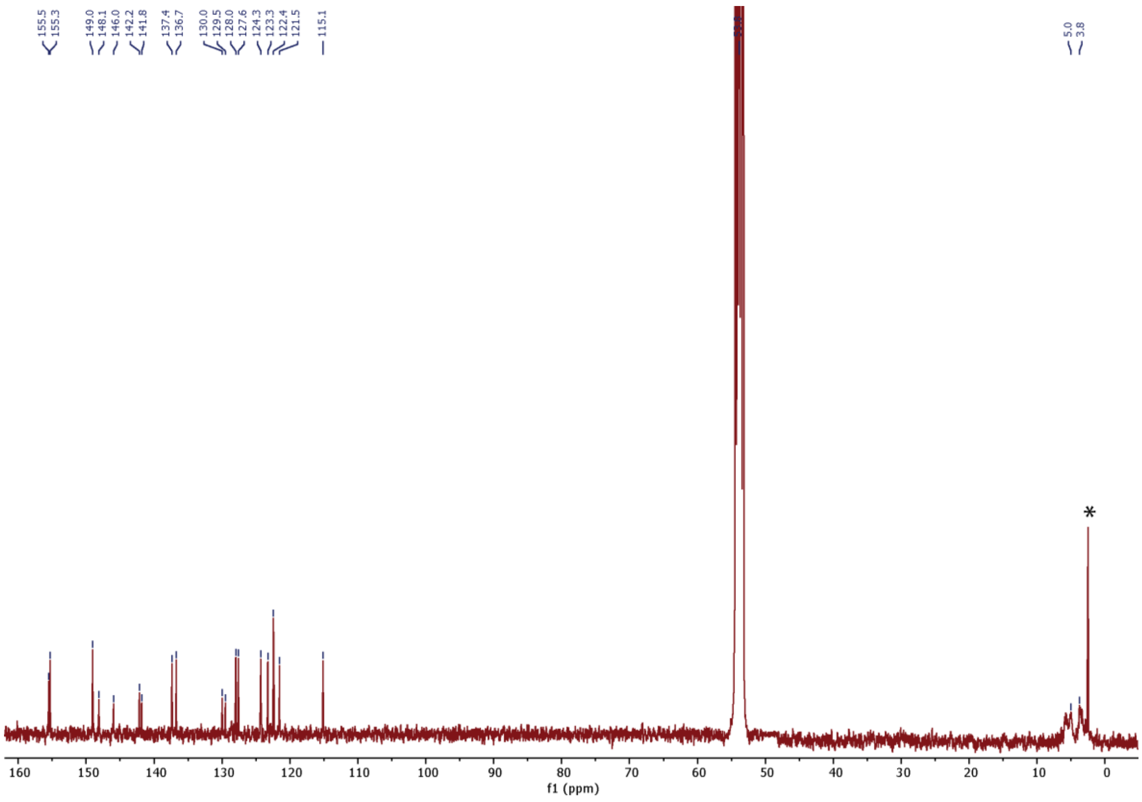

Figure S14. ^{1}H (top, 400.5 MHz) and $^{13}C\{^{1}H\}$ (bottom, 100.7 MHz) NMR spectra (CD_2Cl_2, 298 K) of $[Ru_2\{\mu_{Sn}$-κ^3*Sn,N,N'*-Sn(hmds)(bqfam)$\}(CO)_6]$ (**6b**) (* = Hhmds).

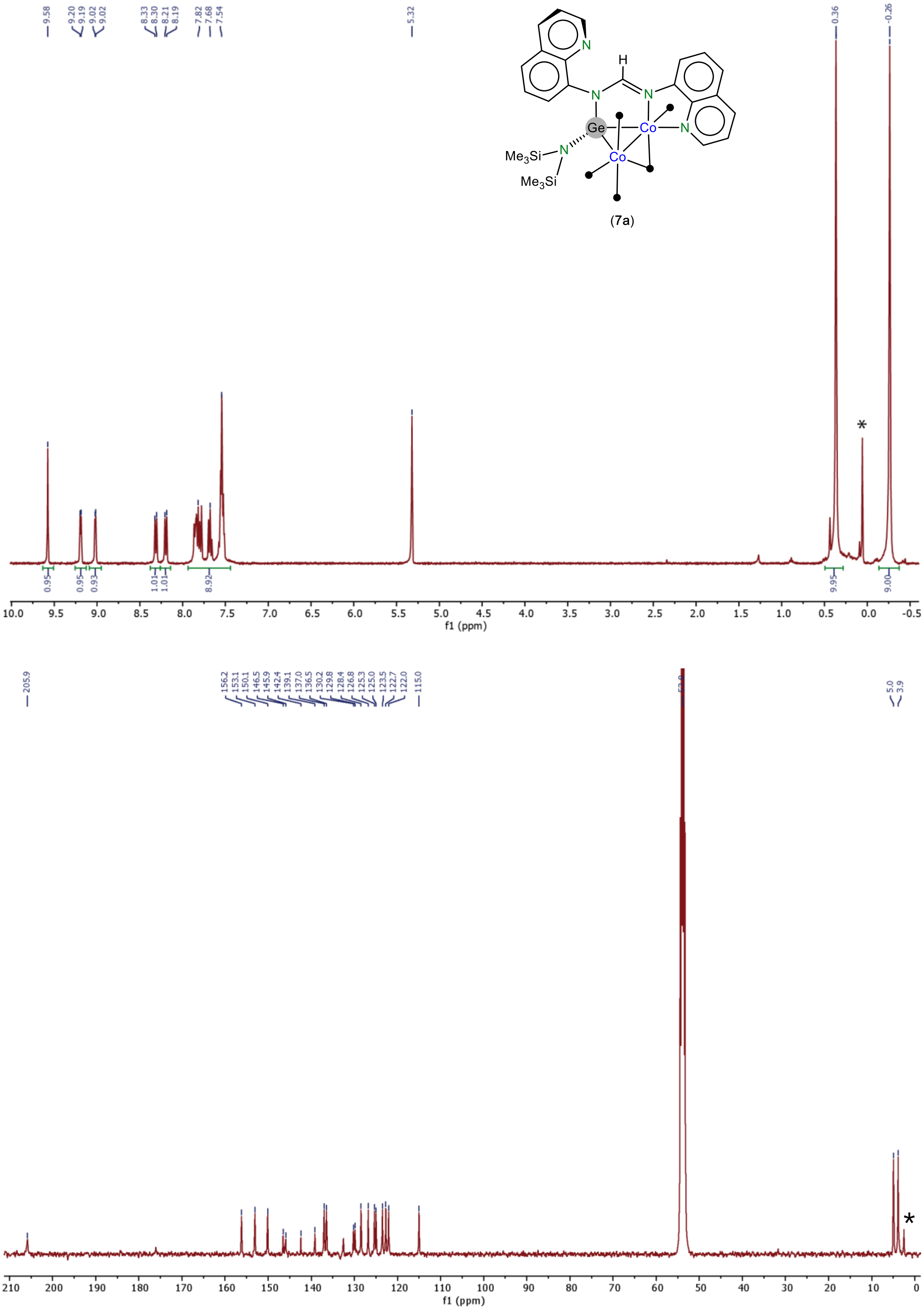

Figure S15. ^{1}H (top, 400.5 MHz) and ^{13}C{^{1}H} (bottom, 100.7 MHz) NMR spectra (CD_2Cl_2, 298 K) of [Co_2{μ_{Ge}-κ^3*Ge,N,N'*-Ge(hmds)(bqfam)}(μ-CO)$(CO)_4$] (**7a**). (* = Hhmds)

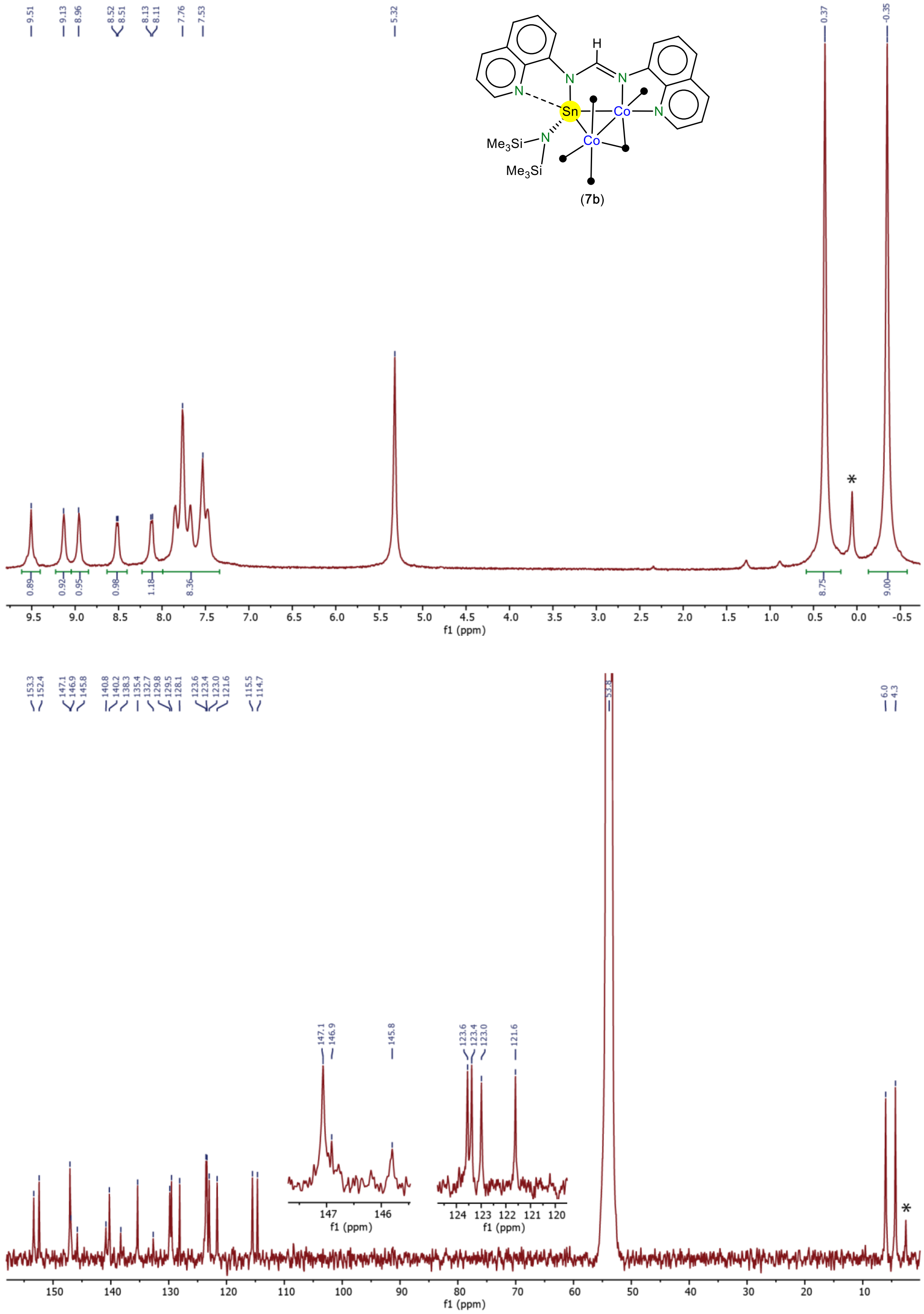

Figure S16. ^{1}H (top, 400.5 MHz) and ^{13}C{^{1}H} (bottom, 100.7 MHz) NMR spectra (CD_2Cl_2, 298 K) of [Co_2{μ_{Sn}-κ^3*Sn,N,N'*-Sn(hmds)(bqfam)}(μ-CO)$(CO)_4$] (**7b**) (* = Hhmds).

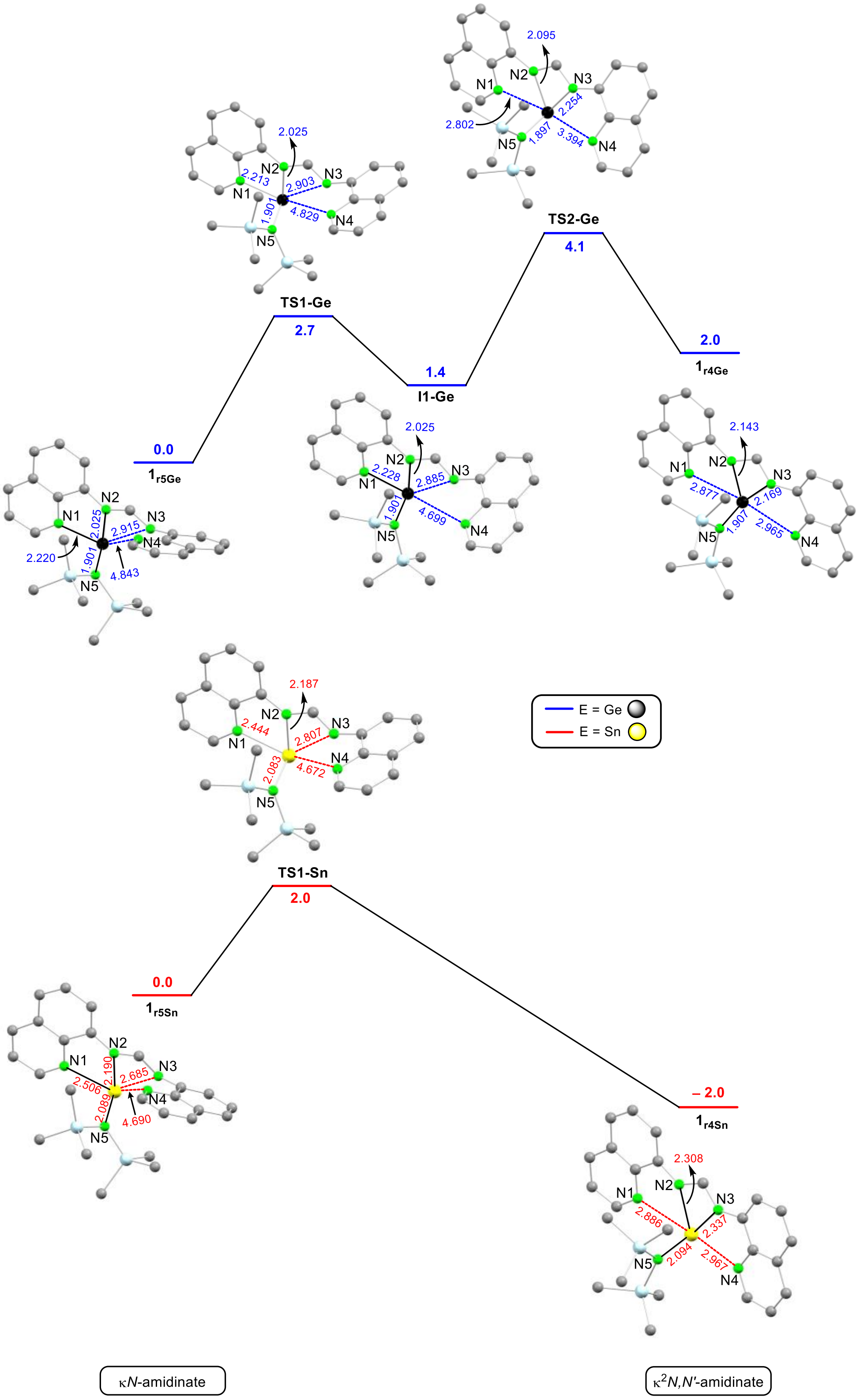

Figure S17. DFT-Calculated (wB97xd/SDD$_{(Ge,Sn)}$/cc-pVDZ) energy profile for the κ*N*-amidinate (**1**$_{\mathbf{r5E}}$) to κ^2*N,N'*-amidinate (**1**$_{\mathbf{r4E}}$) interconversion for E = Ge, Sn. Gibbs energies (CPCM-toluene) are given in kcal mol^{-1}. Interatomic distances are given in angstroms (Å).

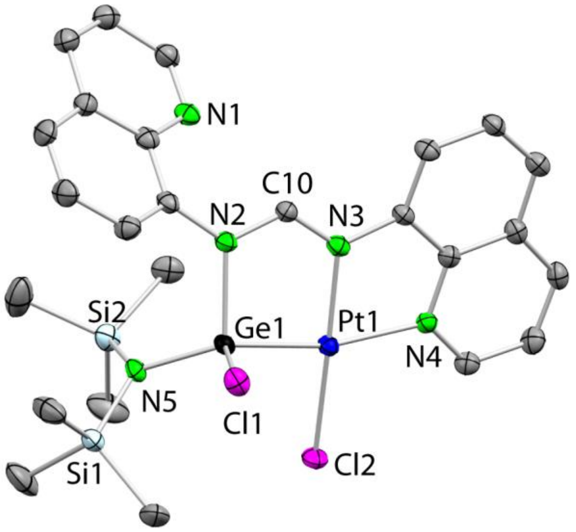

Figure S18. SCXRD molecular structure of **5a** (30% displacement ellipsoids, H atoms omitted for clarity). Selected interatomic distances (Å) and angles (º): Pt1–Ge1 2.2943(6), Pt1–Cl2 2.303(1), Pt1–N3 2.005(4), Pt1–N4 2.103(4), Ge1–N2 1.985(4), Ge1–N5 1.824(4), Ge1–Cl1 2.217(1), N2–C10 1.328(6), N3–C10 1.317(7); N3–Pt1–N4 81.2(2), N3–Pt1–Ge1 85.1(1), N4–Pt1–Ge1 166.2(1), N3–Pt1–Cl2 177.3(1), N4–Pt1–Cl2 98.5(1), Ge1–Pt1–Cl2 95.27(4), N3–C10–N2 122.8(5), N5–Ge1–N2 109.1(1), N5–Ge1–Cl1 105.5(1), N2–Ge1–Cl1 98.1(1), N5 Ge1 Pt1 130.9(1), N2–Ge1–Pt1 95.2(1), Cl1–Ge1–Pt1 112.76(4).

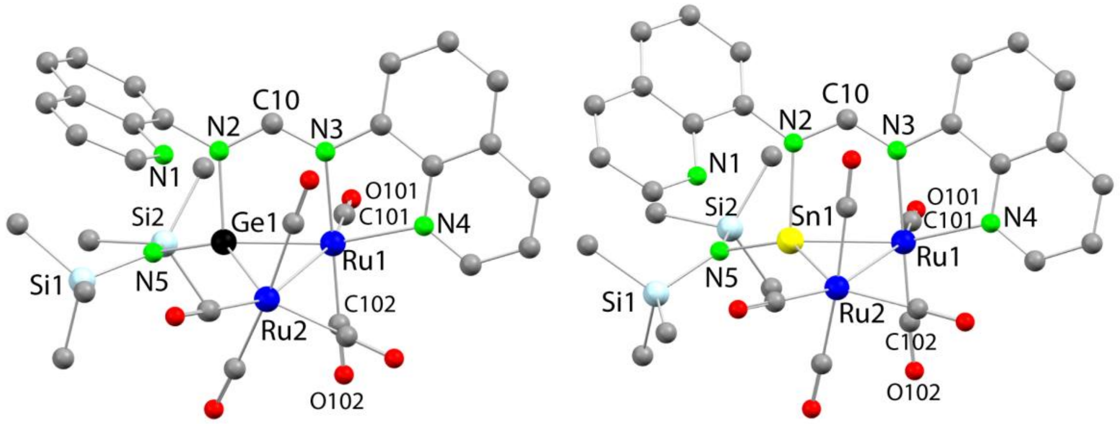

Figure S19. DFT-optimized (wB97xd/SDD$_{(Ge,Sn,Ru)}$/cc-pVDZ) structures of **6a** (left) and **6b** (right). H atoms omitted for clarity. Selected interatomic distances (Å) and angles (º): **6a**: Ru1–Ge1 2.394, Ru1–Ru2 3.011, Ru1–N3 2.145, Ru1–N4 2.205, Ge1–N2 2.015, Ge1···N1 3.689, Ge1–N5 1.856, Ge1–Ru2 2.526; N2–C10 1.320, N3–C10 1.321; N3–C10–N2 121.38, N5–Ge1–N2 100.55, N5–Ge1–Ru2 132.83, N2–Ge1–Ru2 110.77, N5–Ge1–Ru1 136.83, N2–Ge1–Ru1 95.09, Ru1–Ge1–Ru2 75.40. **6b**: Ru1–Sn1 2.560, Ru1–Ru2 3.130, Ru1–N3 2.152, Ru1–N4 2.202, Sn1–N2 2.209, Sn1···N1 3.044, Sn1–N5 2.024, Sn1–Ru2 2.662, N2–C10 1.324, N3–C10 1.320; N3–C10–N2 123.11, N5–Sn1–N2 100.65, N5–Sn1–Ru2 140.28, N2–Sn1–Ru2 109.14, N5 Sn1 Ru1 133.47, N2–Sn1–Ru1 89.34, Ru1–Sn1–Ru2 73.63.
